# Antibiotic‐induced effects on scaling relationships and on plant element contents in herbs and grasses

**DOI:** 10.1002/ece3.4168

**Published:** 2018-06-02

**Authors:** Vanessa Minden, Bernhard Schnetger, Gesine Pufal, Sara D. Leonhardt

**Affiliations:** ^1^ Landscape Ecology Group University of Oldenburg Oldenburg Germany; ^2^ Department of Biology, Ecology and Biodiversity Vrije Universiteit Brussel Brussels Belgium; ^3^ Inorganic Analytical Section University of Oldenburg Oldenburg Germany; ^4^ Nature Conservation and Landscape Ecology University of Freiburg Freiburg Germany; ^5^ Department of Animal Ecology and Tropical Biology University of Würzburg Würzburg Germany

**Keywords:** antibiotics, homeostasis, scaling relationships, standardized major axis regression, tissue nutrient contents

## Abstract

Plant performance is correlated with element concentrations in plant tissue, which may be impacted by adverse chemical soil conditions. Antibiotics of veterinary origin can adversely affect plant performance. They are released to agricultural fields via grazing animals or manure, taken up by plants and may be stored, transformed or sequestered by plant metabolic processes. We studied the potential effects of three antibiotics (penicillin, sulfadiazine, and tetracycline) on plant element contents (macro‐ and microelements). Plant species included two herb species (*Brassica napus* and *Capsella bursa‐pastoris*) and two grass species (*Triticum aestivum and Apera spica‐venti*), representing two crop species and two noncrop species commonly found in field margins, respectively. Antibiotic concentrations were chosen as to reflect in vivo situations, that is, relatively low concentrations similar to those detected in soils. In a greenhouse experiment, plants were raised in soil spiked with antibiotics. After harvest, macro‐ and microelements in plant leaves, stems, and roots were determined (mg/g). Results indicate that antibiotics can affect element contents in plants. Penicillin exerted the greatest effect both on element contents and on scaling relationships of elements between plant organs. Roots responded strongest to antibiotics compared to stems and leaves. We conclude that antibiotics in the soil, even in low concentrations, lead to low‐element homeostasis, altering the scaling relationships between roots and other plant organs, which may affect metabolic processes and ultimately the performance of a plant.

## 1 INTRODUCTION

Element contents of plant tissue have been shown to correlate with the performance of individual plants as well as to influence the structure and function of entire ecosystems (Güsewell, 2004; Sardans, Rivas‐Ubach, & Peñuelas, 2012; Olde Venterink & Güsewell, 2010). Carbon (C), nitrogen (N), and phosphorus (P) are three of the most important elements for plants, and their concentration in plant biomass is directly related to the plant's performance. For example, fast‐growing species have a high biomass P:C ratio and a low biomass N:P ratio, due to higher production of P‐rich ribosomal RNA relative to N‐rich chloroplasts (Growth Rate Hypothesis, see Ågren, 2004; Elser et al., 2000; Sterner & Elser, 2002). In total, plants require about 30 different elements, some in large quantities (macroelements, e.g., H, C, N, O, P, S, K, Ca), others in small quantities (microelements, e.g., Mn, Zn, Fe, Mo) (Ågren, 2008). These elements are allocated to distinct plant organs depending on their respective functions. For example, metabolically active leaves show higher N:P ratios than supporting structures like stems and are generally richer in P, K (potassium), and S (sulphur) than stems, whereas stems show higher C:N ratios than other plant organs (Abrahamson & Caswell, 1982; De Deyn, Cornelissen, & Bardgett, 2008; Güsewell, 2004).

Across and within plant organs, different elements are highly correlated. Kerkhoff, Fagan, Elser, and Enquist (2006) showed that scaling relationships (i.e., trends of allometry or trade‐offs between physical or chemical properties, like mass, size and concentrations) of elements change between organs. The authors focused on N and P content and applied the term “structural” to group stems and roots, and “metabolic” to group leaves and diaspores. Scaling of plant elements among organs of one group (i.e., among stems and roots, or among leaves and diaspores, respectively) yielded isometric relationships (slopes ~1, i.e., constant increase of elements in compared plant organs within one group), for example root N versus stem N or leaf P versus diaspore P. Scaling relationships between plant organs of the separate groups were anisometric (for example, stem N vs. diaspore N or root P vs. leaf P). Anisometric scaling describes a stronger (or less) strong increase in an element of one organ compared to that of another organ. The authors concluded that there was a common set of rules in the partitioning of elements among plant organs, independent of plant functional groups (here: woody species and herbs).

It is notable that internal plant element concentrations are dependent on the external availability of elements (Frost, Evans‐White, Finkel, Jensen, & Matzek, 2005). For example, as response to high external nutrient supplies, primary producers may maximize protein synthesis and growth as competitive strategy, which results in increased biomass element:C ratios (Ågren, 2008; Matzek & Vitousek, 2009). On the other hand, growth and biomass element:C ratios are decreased and nutrients are more effectively used by plants in response to low nutrient conditions (Vitousek, 1982).

Besides low soil nutrient availability, other “adverse chemical soil conditions” (Marschner, 2012) influencing a plants' element concentration are soil acidity (in mineral soils accompanied by aluminum toxicity, see George, Horst, & Neumann, 2012), salt (Minden & Kleyer, 2014), heavy metals (Ghanbarizadeh & Nejad, 2012; Zehra, Arshad, Mahmood, & Waheed, 2009), and pharmaceuticals used in modern medicine (Bártíkova, Podlipná, & Skálová, 2016; der Beek et al., 2016; Kapusta & Godzik, 2013). Among the latter, veterinary antibiotics have attracted the attention of the scientific community due to their effects on biotic and abiotic nontarget organisms. Antibiotics are typically used to kill or inhibit the growth of bacteria for preventing or treating diseases or as growth promoters to increase food production (Du & Liu, 2012; Kumar, Gupta, Chander, & Singh, 2005). Although the use of antibiotics in the EU as livestock growth promoter has been banned since 1998 (CEC, 1998a, 1998b), sales' reports indicate the use of almost 8,500 t of veterinary antibiotics in the EU/EEA (European Economic Area) in 2011 (European Medicines Agency, 2013). The most frequently used antibiotics are *β*‐lactams, sulphonamides, tetracyclines, and macrolides (Du & Liu, 2012; Grave, Torren‐Edo, & Mackay, 2010; Kools, Moltmann, & Knacker, 2008). All of them can subsequently enter the environment through fertilization of soils with manure of livestock animals or through grazing animals. From the soil, they may be transported to ditches, streams, and rivers via runoff (Burkhardt, Stamm, Waul, Singer, & Muller, 2005; Kay, Blackwell, & Boxall, 2005; Stoob, Singer, Mueller, Schwarzenbach, & Stamm, 2007), to groundwater via leaching (Blackwell, Kay, & Boxall, 2007) or may directly be ingested by (nontarget) organisms (Boxall et al., 2006).

Antibiotics in the environment have been recognized as a serious threat to nontarget organisms as well as the entire ecosystem, and have been grouped, together with other pharmaceuticals and personal‐care products, in a new group of chemicals termed “contaminants of emerging concern” (Bartrons & Peñuelas, 2017; Hyland, Blaine, Dickenson, & Higgins, 2015). Once released into the environment, they possibly impact on the development of multi‐resistant bacteria, with detrimental effects on human health as well as on the performance of naturally occurring nontarget species (Bártíkova et al., 2016; Jechalke, Heuer, Siemens, Amelung, & Smalla, 2014; Kumar, Lee, & Cho, 2012; Minden, Deloy, Volkert, Leonhardt, & Pufal, 2017). In plants, antibiotics can, among other effects, delay germination, reduce chlorophyll content and growth, and affect bioaccumulation (see summarizing tables in Bártíkova et al., 2016; Carvalho, Basto, Almeida, & Brix, 2014; Minden et al., 2017; Puckowski et al., 2016). It is notable that the effects of antibiotics on element contents of plants have received much less attention. To our knowledge, only one study has hitherto investigated effects of sulfadiazine on the C:N and K:Ca ratios of willow and maize plants, and found significantly lower C:N and K:Ca ratios in high antibiotic treatments (Michelini, Reichel, Werner, Ghisi, & Thiele‐Bruhn, 2012). However, this study used concentrations (200 μg/g soil) that are much higher than typically found in agricultural soils (0.006–500 μg/kg soil, Thiele‐Bruhn, 2003). The use of unnaturally high concentrations of antibiotics has recently been recognized as major drawback in relating results to in vivo situations (Bártíkova et al., 2016).

This study focuses on the effects of “in vivo concentrations” of veterinary antibiotics on element contents of plants. We tested how three different antibiotics (i.e., penicillin, tetracycline, and sulfadiazine) differing in their action modes affected element contents of four plant species, including crop (*Brassica napus* and *Triticum aestivum*) and noncrop (*Capsella bursa‐pastoris* and *Apera spica‐venti*) species. A previous study on antibiotic‐effects on the performance of these plant species revealed significant effects on chlorophyll content, growth rates, and biomass allocation on the target organisms (Minden et al., 2017). Both crop species (*B. napus* and *T. aestivum*) belong to the most commonly grown crops worldwide (FAO, 2016; Leff, Ramankutty, & Foley, 2004) and are thus highly likely exposed to antibiotics due to fertilization of crop fields with slurry or manure. The noncrop species (*C. bursa‐pastoris* and *A. spica‐venti*) are commonly found along most crop field margins in Germany and are likely unintentionally exposed to antibiotic‐charged manure applied to fields (Ellenberg & Leuschner, 2010). We applied concentrations of antibiotics as previously reported for grasslands (from now on referred to as in vivo concentrations, Thiele‐Bruhn, 2003) to plants grown in greenhouses and measured macro‐ and micronutrients (N, P, K, C, Ca, S, Cu, Mg, Fe, Mn, Na) in fully developed plant individuals.

We investigated (a) whether in vivo concentrations of antibiotics generally affected element contents of plants and (b) whether plant responses differed between antibiotics, antibiotic concentrations and plant organs. Here, we expected roots to be most affected as shown by previous studies on antibiotics (Migliore, Rotini, Cerioli, Cozzolino, & Fiori, 2010; Pierattini, Francini, Raffaelli, & Sebastiani, 2016). We further tested whether scaling relationships between elements of different plant organs were either concurrent or discontinuous with the patterns of isometry and anisometry found by Kerkhoff et al. (2006) for plants grown under natural conditions.

## 2 MATERIALS AND METHODS

### 2.1 Selected plant species

The experiment was carried out with two crop species and two noncrop species, with one representative of either group belonging to the family of Brassicaceae (*B. napus* L. [summer rapeseed] and *C. bursa‐pastoris* L. [shepherd's purse]) or Poaceae (*T. aestivum* L. [wheat] and *A. spica‐venti* L. [loose silky‐bent]). Our choice allowed for a comparison between crop plants and noncrop plants within the functional groups of herbs (Brassicaceae) and grasses (Poaceae), respectively, and minimized a potential bias associated with phylogenetic relationships or differences in life‐history or dispersal mode (congeneric or phylogenetic approach, Burns, 2004; van Kleunen, Weber, & Fischer, 2010). All species were annuals.

Seeds of the plants were ordered in April 2015 from Rieger‐Hofmann^®^, Germany (*C. bursa‐pastoris*,* A. spica‐venti*), Sämereien Jehle, Germany (*B. napus*), and Botanik Sämereien, Switzerland (*T. aestivum*).

### 2.2 Selected antibiotics and their modes of action

The three antibiotics used in our study are penicillin G sodium salt (C_16_H_17_N_2_NaO_4_S), sulfadiazine (C_10_H_10_N_4_O_2_S), and tetracycline (C_22_H_24_N_2_O_8_). They are the most commonly sold antibiotic compound classes used for food‐producing animal species in Europe, with 37%, 23%, and 11% of sold antibiotics, respectively (European Medicines Agency, 2013; Tasho & Cho, 2016). As they are polar (with logKW < 3) they likely accumulate in plant tissue (Trapp & Eggen, 2013).

In general, both biodegradation and modes of action differ between the different types of antibiotics. Half‐life for sulfadiazine is 50 days, whereas soil‐stability for penicillin and tetracycline ranges from 40 days to 2 years, respectively (Christian et al., 2003; Hamscher, Pawelzick, Hoper, & Nau, 2005; Kumar, Gupta, Baidoo, Chander, & Rosen, 2005). Penicillin G (*β*‐lactam antibiotic) inhibits the biosynthesis of peptidoglycan during microbial cell division and thus cell wall synthesis (Hammes, 1976; Miller, 2002). Sulfadiazine inhibits the growth of bacteria without destroying them (bacteriostasis) (Henry, 1944). Tetracycline is an anti‐infective agent inhibiting protein synthesis by preventing the attachment of aminoacyl‐t‐RNA to the ribosomal acceptor (Chopra & Roberts, 2001). For a complete list of known examples for effects of these antibiotics on various plants species see Minden et al. (2017).

### 2.3 Experimental design

Plants were treated with 1, 5, and 10 μg antibiotic/L for penicillin (P1, P5, and P10), sulfadiazine (S1, S5, and S10), and tetracycline (T1, T5, and T10), respectively, by adding antibiotics to soil water. In addition, we applied one nitrogen treatment (N10, see below) and one control treatment (C) (distilled water). To avoid confounding effects of mixtures of antibiotics, these compounds were added as separate treatments.

Converted to the amount of sand in the pots, treatments corresponded to 0.038, 0.19, and 0.38 μg/kg sand (see description of greenhouse experiment below). Antibiotics were ordered at Alfa Aesar (Karlsruhe, Germany). Antibiotic solutions were prepared by dissolving 1 mg of antibiotic in 1 L distilled water, before adding up 1 ml (5 and 10 ml) of removed solution to distilled water for a final volume of 1 L; pHs of all solutions were 5.5.

As all antibiotics used contain a nitrogen group (i.e., one molecule penicillin contains 7.8% N, tetracycline 6.3% N and sulfadiazine 22.4% N), we included one nitrogen (N‐) treatment to differentiate between potential plant responses to antibiotics and/or to nitrogen provided by antibiotic degradation. We chose the highest amount of nitrogen provided by the antibiotics treatments (i.e., sulfadiazine treatment) as applied in the 10 μg/L treatment. Thus, 13.58 mg NaNO_3_ were diluted in 1 L distilled water and 1 ml of this solution was further diluted with 1 L distilled water.

Macro‐ and micronutrients (N, P, K, Ca, S, Cu, Mg, Fe, B, Mn, Zn, Mo) were evenly applied to each experimental pot (5 ml solution/week). Nitrogen was applied as NaNO_3_ and phosphorus as NaH_2_PO_4_. Composition of nutrient solutions followed Güsewell (2004), pH was adjusted to 6.

### 2.4 Greenhouse experiment

Ten individuals per plant species were exposed to each treatment, summing up to 110 individuals per species and 440 individuals in total. Plants were raised from seeds in germination pots with germination soil (Gartenkrone, Germany). In June 2015, about 3 weeks after sowing, individual plants were planted in 400 ml pots filled with quartz sand (Vitakraft, Germany), *B. napus* was planted in 2‐L pots. We used quartz sand instead of potting soil to guarantee a homogenous substrate for all treatments and thus prevent variation in soil‐related factors (e.g., water‐holding capacity) across pots. Also, antibiotics are organic compounds with a tendency to adsorb to soil particles, depending on soil pH, soil organic matter, and soil minerals (Tasho & Cho, 2016; Tolls, 2001). Using quartz sand, we restrained adsorption to soil organic matter, which can be strong (Thiele, 2000).

We mixed 25 ml of antibiotic and/or nitrogen solution with the sand before the seedlings were planted (125 ml for the 2‐L pots). This volume was equivalent to the quantity held back by quartz sand without draining. To avoid leaching of the antibiotics from pots, distilled water was filled into saucers only when needed. Nutrient solutions were provided once a week for 8 weeks in total. Control treatments received only distilled water and nutrients. Pots were randomly distributed in the greenhouse and shuffled once a week.

At the end of the experiment (i.e., after 8 weeks), plant individuals were harvested and separated into leaves, stems, and roots, dried at 70°C for 72 hr and weighed. Dead leaves were sorted and excluded from further analysis. Dried material of each organ of each harvested plant individual was ground in a planetary mill at 300–400 revolutions (“pulverisette 7”; Fritsch, Idar‐Oberstein, Germany). For C:N analysis, each sample was further dried at 105°C for 4–5 hr. Then, 2–3 mg of material were placed into tin tubes (0.1 mg precision balance CP 225 D; Sartorius, Goettingen, Germany) and analyzed using a CHNS Analyser Flash EA (Thermo Electron Corp., Waltham, MA, USA) following Allen (1989). All other elements (P, K, Ca, S, Cu, Mg, Fe, Mn, Na) were analyzed using optical emission spectrometry with inductively coupled plasma (ICP‐OED: iCAP 6000 radial plasma view, Thermo Scientific), for which 8–10 mg ground material were digested with nitric acid and hydrogen peroxide. For every element, we used at least two spectral lines to identify interferences. A solution with several internal standards was added to sample and calibration solutions for improving precision. Allocation of internal standard spectral lines to the analyte lines was chosen to match the product of ionization potential and energy of the spectral line. All values of plant elements refer to mg/g. For primary functions of elements see Supporting Information Appendix 1.

### 2.5 Statistical analysis

All statistical analyses were carried out with the computer software R (R Core Team, 2014). Packages used were geoR (Ribeiro & Diggle, 2015), car (Fox & Weisberg, 2011), nortest (Gross & Ligges, 2015), and smatr (Warton, Duursma, Daniel, & Taskinen, 2012).

We first tested for significant differences between the nitrogen treatment and the control treatment, with the hypothesis that nitrogen addition in such small amounts should not have an effect on plant elements. Indeed, analyses yielded no differences, and data of the nitrogen treatment and the control treatment were thus pooled into one control treatment in subsequent analyses.

To test for effects of plant species identity, plant organs, type of antibiotics and their concentrations on the response variables (i.e., plant element content), multifactorial analyses of variance (ANOVA) were carried out. Factors were species (four levels: *B. napus*,* C. bursa‐pastoris*,* T. aestivum* and *A. spica‐venti*), plant organ (three levels: leaves, stems and roots), antibiotics (three levels: penicillin, sulfadiazine and tetracycline), and concentration (four levels: 0, 1 5, 10 μg/L) as well as their interactions. Level 0 of the concentration treatment refers to the pooled nitrogen and the control treatment (i.e., no application of antibiotics).

As our main research aim was to investigate whether in vivo concentrations of antibiotics generally affect element contents of plants, we subsequently performed one‐way ANOVA between the control and antibiotic treatments (P1, P5, P10, S1, S5, S10, T1, T5, and T10, respectively) for each element separated for plant species and plant organ. Tukey's honest significant differences tests with false discovery rate correction was used to assess differences between individual groups (i.e., between control group and antibiotic treatments) (Benjamini & Hochberg, 1995; Noble, 2009). For all tests, element contents were transformed (log, boxcox) if necessary to meet the assumptions of parametric testing.

To describe bivariate relationships between element contents of different plant organs, we used standardized major axis regression (SMA). This analysis summarizes the relationship between two variables by minimizing the residuals in both variables (Kerkhoff et al., 2006; Warton, Wright, Falster, & Westoby, 2006), rather than predict one variable from the other (e.g., *Y* from *X*), which would be best described by ordinary least squares regression (Niklas, 2006). To test for significant deviations from isometric scaling (slope ~1) between the element contents of the plant organ combinations, we used the function sma(*y*~*x*, slope.test=1) (R package smatr, Warton et al., 2012).

We calculated SMA regressions for the plant organ combinations stem versus leaves, roots versus stems, and roots versus leaves. We first did this for each element and antibiotic for each of the four plant species separately (plant species‐specific dataset). However, these SMA regression results made it difficult to derive common patterns of isometry and anisometry (see slopes in Supporting Information Appendix 2), probably because the range of each element for each species was too narrow to result in a significant relationship between plant organs (see means and standard deviations of each element in each organ for each species in Supporting Information Appendix 3). To test our hypothesis that patterns of isometry and anisometry as described by Kerkhoff et al. (2006) may be disrupted by antibiotics, we, therefore, merged the plant species‐specific datasets to one pooled dataset containing all four plant species and repeated the analyses.

## 3 RESULTS

**Figure 1:**
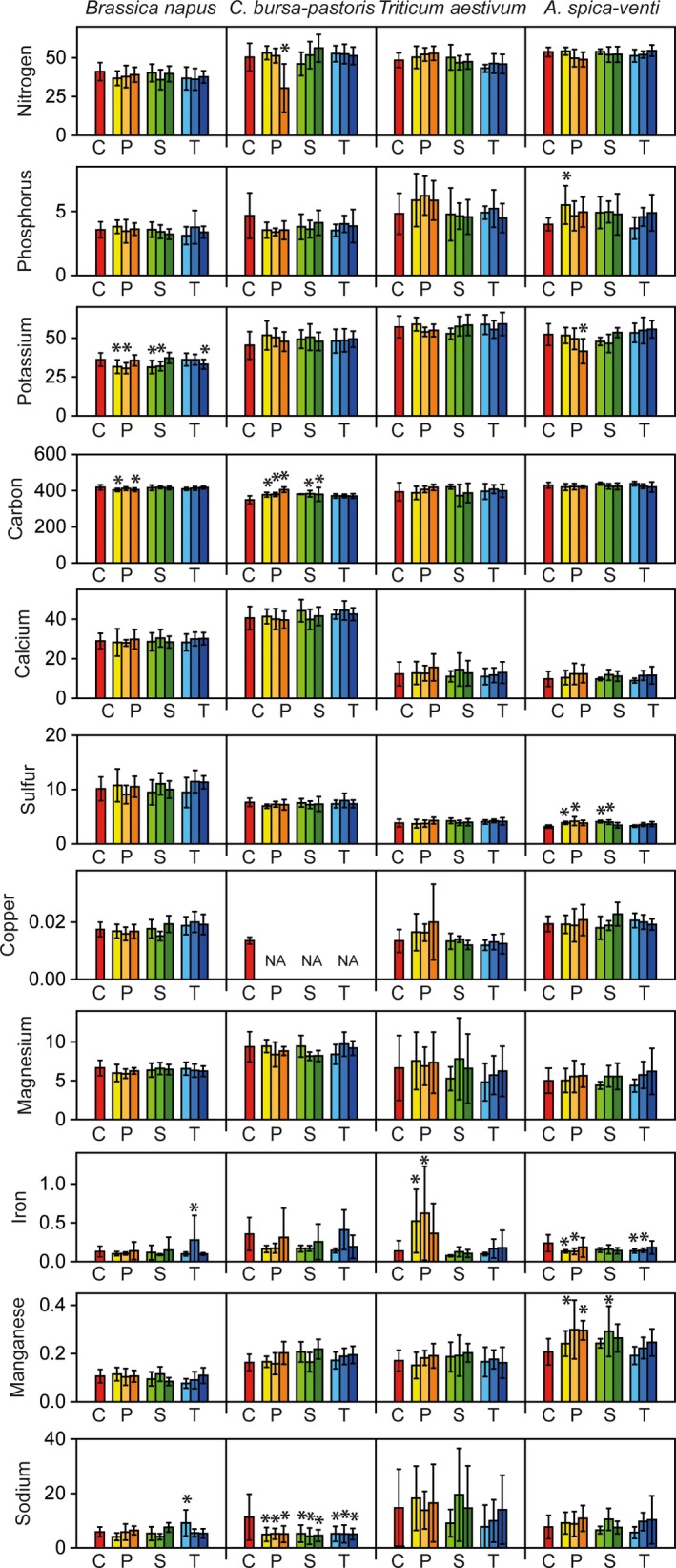
Effects of antibiotics on plant elements (mg/g) concentrations in leaves of *Brassica napus*,* Capsella bursa‐pastoris*,* Triticum aestivum*, and *Apera spica‐venti*. Red bars show control treatments (C), antibiotics treatments were penicillin (P, yellow bars), sulfadiazine (S, green bars), and tetracycline (T, blue bars), in concentrations of 1, 5, and 10 μg/L. Values presented are the means of 10 replicates with the standard deviations shown in vertical bars (20 replicates for the control treatment). Asterisks indicate significant differences between control treatment and antibiotic treatment at *p* < 0.05, according to Tukey HSD test with false discovery rate correction

**Figure 2:**
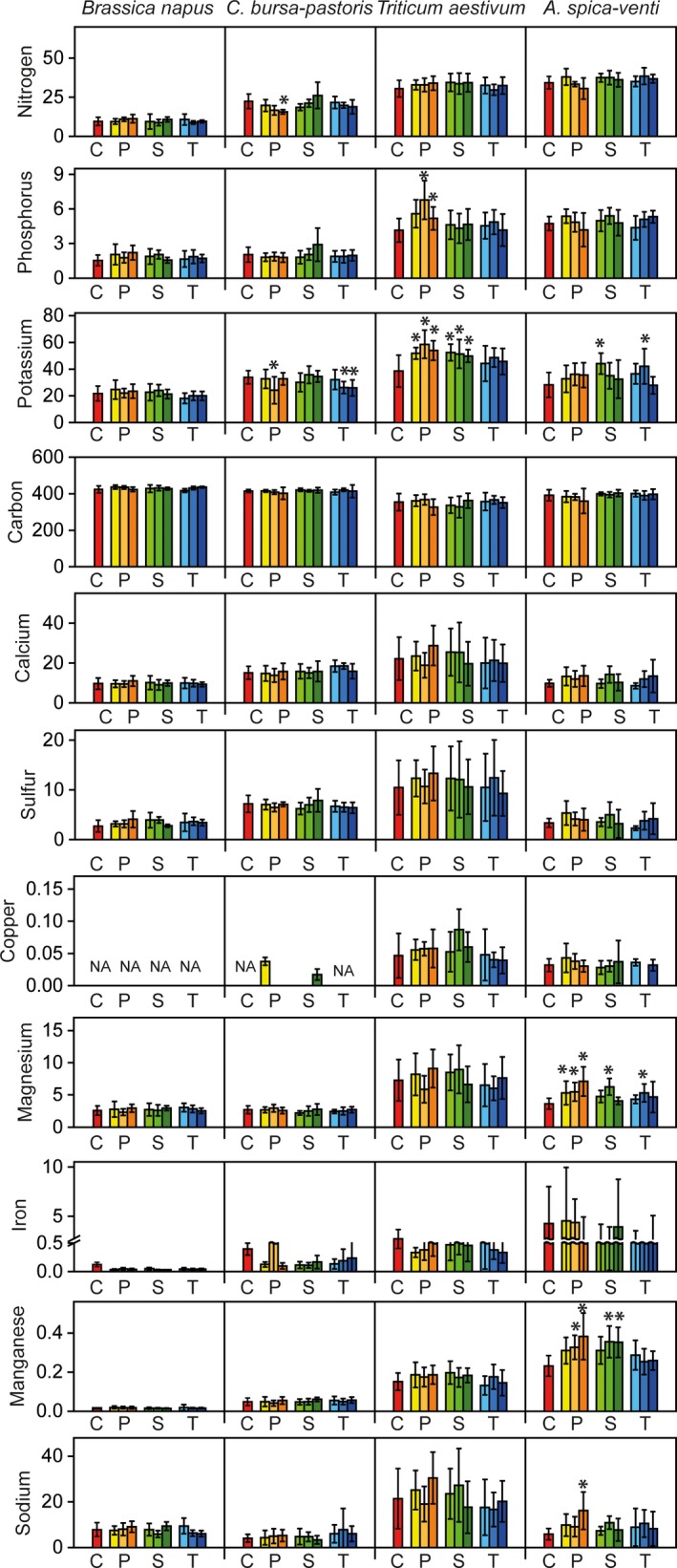
Effects of antibiotics on plant elements (mg/g) concentrations in stems of *Brassica napus*,* Capsella bursa‐pastoris*,* Triticum aestivum*, and *Apera spica‐venti*. Red bars show control treatments (C), antibiotics treatments were penicillin (P, yellow bars), sulfadiazine (S, green bars), and tetracycline (T, blue bars), in concentrations of 1, 5, and 10 μg/L. Values presented are the means of 10 replicates with the standard deviations shown in vertical bars (20 replicates for the control treatment). Asterisks indicate significant differences between control treatment and antibiotic treatment at *p* < 0.05, according to Tukey HSD test with false discovery rate correction

**Figure 3:**
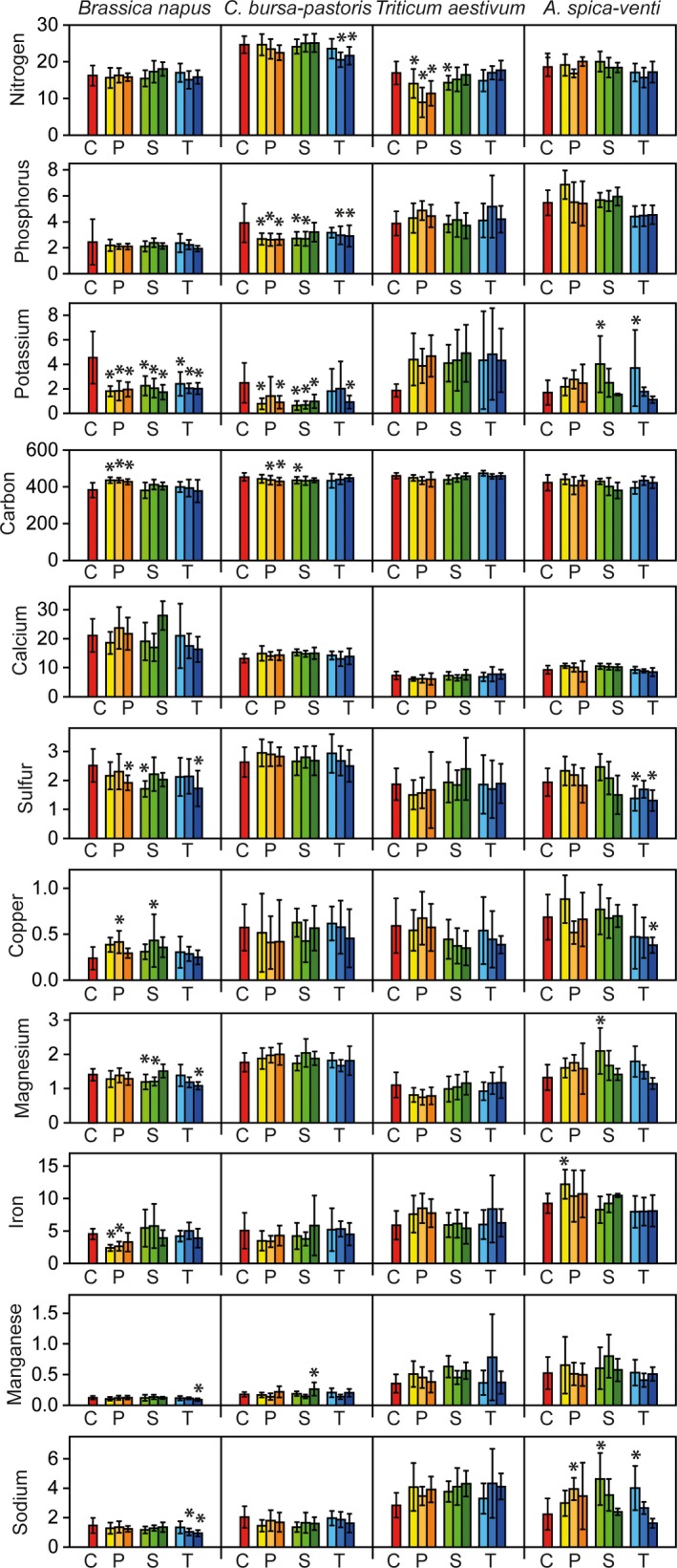
Effects of antibiotics on plant elements (mg/g) concentrations in roots of *Brassica napus*,* Capsella bursa‐pastoris*,* Triticum aestivum*, and *Apera spica‐venti*. Red bars show control treatments (C), antibiotics treatments were penicillin (P, yellow bars), sulfadiazine (S, green bars), and tetracycline (T, blue bars), in concentrations of 1, 5, and 10 μg/L. Values presented are the means of 10 replicates with the standard deviations shown in vertical bars (20 replicates for the control treatment). Asterisks indicate significant differences between control treatment and antibiotic treatment at *p* < 0.05, according to Tukey HSD test with false discovery rate correction

Mean values for N, P, K, Ca, Mg, and S were highest in leaves, second highest in roots and lowest in stems (see Figures 1, 2, and 3, for mean values see Supporting Information Appendix 3). For some elements like N, concentrations were four times higher in leaves than in stems. C was the most abundant element across all species, followed by N and K.

The results of multi‐factor ANOVA analyses showed that responses differed between elements: N, K, C, and Mn significantly responded to the interactions between the four factors species (S), organ (O), antibiotic (A), and concentration (C), while for the remaining plant elements, interactions between two or three factors yielded significant results (see Table 1). Overall, the factors species and organ had the strongest effects on plant elements, followed secondly by antibiotics and their concentrations. However, interactions between antibiotics and concentrations with species and plant organs affected all plant elements but Ca and S. Antibiotic‐effects differed between species (S × A, significant for N, P, C, Cu, Fe) and between plant organs (O × A, significant for K, Cu). Also, concentration‐effects differed between species (S × C, significant for N, P, K, Cu, Mg, Fe, Mn, and Na) and between plant organs (O × C, significant for N, P, K, Cu, Mg, Na).

**Table 1:** *F*‐values, degrees of freedom (*df*), and significance levels for multi‐factor ANOVA analyses testing the effects of plant species (S), organ (O), antibiotic (A), and concentration (C) on elements in plant tissues (mg/g)

	*df*	N	P	K	C	Ca	S	Cu	Mg	Fe	Mn	Na
Species (S)	3	**822.10** ***	**708.44** ***	**477.53** ***	**15.32** ***	**592.60** ***	**124.50** ***	**106.96** ***	**65.72** ***	**201.06** ***	**474.98** ***	**178.81** ***
Organ (O)	2	**5,686.12** ***	**257.11** ***	**7,754.71** ***	**193.49** ***	**714.36** ***	**799.51** ***	**1,009.22** ***	**1,514.32** ***	**2,161.31** ***	**453.84** ***	**309.99** ***
Antibiotic (A)	2	**5.79** **	**3.50** *	0.73	0.01	1.03	0.50	2.52	1.74	1.31	**4.40** *	2.09
Conc. (C)	3	**5.84** ***	1.31	**3.50** *	0.37	0.42	0.50	**3.34** *	0.41	2.19	**7.43** ***	0.66
S × O	6	**244.96** ***	**101.54** ***	**131.08** ***	**198.44** ***	**471.97** ***	**309.60** ***	**29.25** ***	**154.22** ***	**76.64** ***	**76.69** ***	**44.56** ***
S × A	6	**3.20** **	**4.77** ***	1.57	**2.35** *	0.99	0.54	**2.16** *	1.01	**6.37** ***	2.04	1.60
O × A	4	0.06	0.42	**5.57** ***	1.26	0.45	0.74	**2.60** *	0.39	0.30	1.28	0.50
S × C	9	**2.02** *	**8.04** ***	**7.87** ***	1.84	1.39	0.67	**2.78** **	**2.62** **	**2.69** **	**4.60** ***	**3.05** **
O × C	6	**4.29** ***	**3.01** **	**8.72** ***	0.50	0.93	1.27	**2.12** *	**2.13** *	2.01	1.42	**2.30** *
A × C	6	**6.92** ***	1.80	0.61	0.82	0.76	1.48	0.69	2.07	0.65	2.03	1.84
S × O × A	12	**3.97** ***	0.87	1.64	**2.33** **	0.95	0.20	1.38	0.83	**3.40** ***	0.54	0.59
S × O × C	18	**2.89** ***	1.28	**5.53** ***	**3.97** ***	0.49	0.75	1.59	1.09	**2.85** ***	**2.98** ***	1.26
S × A × C	18	**4.39** ***	**2.58** ***	1.20	1.14	1.33	0.72	1.09	1.15	**2.66** ***	**3.15** ***	**1.81** *
O × A × C	12	**1.92** *	0.39	**2.85** ***	**2.35** **	**1.94** *	0.89	0.74	1.07	0.90	1.06	0.97
S × O × A × C	36	**2.63** ***	0.64	**1.74** **	**1.62** *	1.00	0.59	1.06	0.85	1.38	**2.07** ***	0.71

Note. Significance levels: *p < 0.05, **p < 0.01, ***p < 0.001, significant results in bold.

Pairwise comparisons between control and antibiotic treatments showed that element contents in roots were most strongly affected by antibiotics, whereas stem element contents were weakly affected (Figures 1, 2, and 3). We found 50 comparisons between control and antibiotic treatments (Tukey HSD tests) to be significant for roots (corresponding to 13% of all tests for this plant organ), 20 for leaves (5% of all tests), and 13 for stems (3%), most of them in the penicillin treatments (Figures 1, 2, and 3). K was most responsive to the treatments, followed by N, C, S, and Cu. It is interesting that the values of element contents were mostly lower in the antibiotic treatments than in the control treatments for the two herb species (for N, K, Fe, and Na), whereas the two grass species showed reverse patterns: for P, K, Mg, and Na, element contents were higher in the antibiotics treatments than in the control treatments for *T. aestivum* and *A. spica‐venti*, respectively (Figures 1, 2, and 3, and Supporting Information Appendix 3).

Table 2 shows the results of SMA regression for the pooled dataset, in which all separate species‐specific datasets were merged. We found significant correlations for element contents between organs for 92 of the 132 tests performed, of which most were detected between roots and stems. The slope of the regression as a measure of the extent of the increase or decrease of one element in two plant organs relative to each other indicates isometric or anisometric scaling relationships between these plant organs. Following the patterns derived from Kerkhoff et al. (2006), we expected the slopes between stems and leaves to be greater than 1 (*α* > 1, with stems on the *y*‐axis and leaves on the *x*‐axis), between roots and stems to be 1 (*α *~ 1) and between roots and leaves to be smaller than 1 (*α* < 1, with roots on the *y*‐axis and leaves on the *x*‐axis). The data supported these expectations in 70% of all significant results for stems versus leaves. Significant slopes for roots versus stems were mainly <1 and for root versus leaves either ~1 or >1.

**Table 2:** Slopes of standardized major axis (SMA) regression, confidence intervals, and correlation coefficients (*r*) for all combinations of plant organs (in the order of *Y* vs. *X*: stem vs. leaf, root vs. stem, and root vs. leaf) for each element across all species (pooled dataset)

		Control (C)	Penicillin (P)	Sulfadiazine (S)	Tetracycline (T)	C	P	S	T
SMA of N									
Stem	Leaf	**3.52 (2.87, 4.32), 0.37**	**2.92 (2.51, 3.39), 0.47**	**3.31 (2.85, 3.85), 0.46**	**2.86 (2.48, 3.30), 0.45**	✓	✓	✓	✓
Root	Stem	·	**−0.71 (−0.87, −0.58), 0.06**	·	·	·	✗	·	·
Root	Leaf	·	·	**1.21 (0.98, 1.50), 0.05**	0.94 (0.78, 1.13), 0.17	·	·	✗	✗
SMA of P									
Stem	Leaf	**2.10 (1.69, 2.62), 0.25**	**1.83 (1.61, 2.08), 0.59**	**1.97 (1.70, 2.29), 0.38**	**2.09 (1.8, 2.43), 0.39**	✓	✓	✓	✓
Root	Stem	0.83 (0.69, 1.00), 0.47	**0.76 (0.67, 0.86), 0.63**	**0.72 (0.64, 0.82), 0.62**	**0.7 (0.62, 0.79), 0.60**	✓	✗	✗	✗
Root	Leaf	**1.60 (1.27, 2.01), 0.15**	**1.49 (1.27, 1.74), 0.34**	**1.51 (1.29, 1.77), 0.37**	**1.51 (1.29, 1.77), 0.34**	✗	✗	✗	✗
SMA of K									
Stem	Leaf	**1.62 (1.29, 2.04), 0.19**	**1.74 (1.49, 2.04), 0.37**	**1.64 (1.42, 1.89), 0.46**	**1.74 (1.52, 1.98), 0.53**	✓	✓	✓	✓
Root	Stem	·	**1.97 (1.63, 2.39), 0.11**	**2.22 (1.84, 2.68), 0.11**	**1.77 (1.47, 2.13), 0.11**	·	✗	✗	✗
Root	Leaf	**−2.80 (−3.41, −2.30), 0.37**	·	·	·	✓	·	·	·
SMA of C									
Stem	Leaf	1.08 (0.84, 1.38), 0.09	·	**1.27 (1.07, 1.51), 0.29**	·	✗	·	✓	·
Root	Stem	−0.98 (−1.24, −0.77), 0.20	·	−0.69 (−0.84, −0.57), 0.11	−1.12 (−1.36, −0.93), 0.11	✓	·	✗	✓
Root	Leaf	−1.11 (−1.41, −0.88), 0.14	−1.01 (−1.23, −0.83), 0.06	−0.94 (−1.16, −0.76), 0.08	−**1.57 (**−**1.91,** −**1.29), 0.05**	✗	✗	✗	✓
SMA of Ca									
Stem	Leaf	·	·	·	**0.65 (0.54, 0.79), 0.05**	·	·	·	✗
Root	Stem	−0.98 (−1.24, −0.77), 0.15	**−1.25 (−1.47, −1.06), 0.37**	−0.96 (−1.14, −0.80), 0.22	·	✓	✗	✓	·
Root	Leaf	**0.65 (0.54, 0.79), 0.40**	0.88 (0.75, 1.03), 0.33	**0.75 (0.64, 0.88), 0.36**	**0.65 (0.56, 0.76), 0.42**	✓	✗	✓	✓
SMA of Cu									
Stem	Leaf	**2.07 (1.46, 2.94), 0.21**	·	·	·	✓	·	·	·
Root	Stem	1.14 (0.83, 1.57), 0.33	**1.66 (1.29, 2.15), 0.20**	·	1.24 (0.86, 1.79), 0.24	✓	✗	·	✓
Root	Leaf	·	**2.33 (1.89, 2.88), 0.19**	·	·	·	✗	·	·
SMA of Mg									
Stem	Leaf	·	**−1.59 (−1.94, −1.31), 0.04**	·	·	·	✗	·	·
Root	Stem	**−0.56 (−0.72, −0.44), 0.09**	**−0.81 (−0.95, −0.68), 0.35**	**−0.58 (−0.68, −0.49), 0.28**	**−0.66 (−0.79, −0.55), 0.12**	✗	✗	✗	✗
Root	Leaf	·	·	·	·	·	·	·	·
SMA of S									
Stem	Leaf	·	**−1.36 (−1.66, −1.12), 0.06**	**−1.35 (−1.63, −1.12), 0.07**	·	·	✗	✗	·
Root	Stem	**−0.45 (−0.58, −0.36), 0.10**	**−0.68 (−0.84, −0.56), 0.12**	·	**0.60 (0.49, 0.73), 0.04**	✗	✗	·	✗
Root	Leaf	**0.60 (0.48, 0.75), 0.20**	0.87 (0.73, 1.05), 0.09	·	**0.80 (0.67, 0.96), 0.13**	✓	✗	·	✓
SMA of Fe									
Stem	Leaf	**3.37 (2.63, 4.32), 0.07**	·	·	·	✓	·	·	·
Root	Stem	**0.22 (0.18, 0.27), 0.40**	**0.39 (0.34, 0.45), 0.55**	**0.34 (0.29, 0.41), 0.18**	**0.29 (0.24, 0.34), 0.25**	✗	✗	✗	✗
Root	Leaf	**0.74 (0.58, 0.93), 0.06**	0.89 (0.74, 1.06), 0.16	·	·	✓	✗	·	·
SMA of Mn									
Stem	Leaf	**3.20 (2.68, 3.81), 0.55**	**2.80 (2.46, 3.18), 0.60**	**2.63 (2.33, 2.97), 0.60**	**2.57 (2.25, 2.93), 0.53**	✓	✓	✓	✓
Root	Stem	**0.55 (0.48, 0.63), 0.70**	**0.59 (0.53, 0.66), 0.70**	**0.63 (0.57, 0.70), 0.75**	**0.66 (0.60, 0.74), 0.72**	✗	✗	✗	✗
Root	Leaf	**1.78 (1.49, 2.12), 0.47**	**1.75 (1.50, 2.04), 0.37**	**1.73 (1.49, 2.01), 0.43**	**1.72 (1.47, 2.01), 0.36**	✗	✗	✗	✗
SMA of Na									
Stem	Leaf	0.96 (0.78, 1.18), 0.33	**1.31 (1.14, 1.51), 0.51**	1.13 (0.98, 1.30), 0.47	1.09 (0.95, 1.24), 0.51	✗	✓	✗	✗
Root	Stem	**0.63 (0.49, 0.80), 0.16**	**0.65 (0.54, 0.78), 0.22**	**0.71 (0.59, 0.85), 0.24**	0.86 (0.72, 1.03), 0.21	✗	✗	✗	✓
Root	Leaf	·	**0.84 (0.71, 0.98), 0.35**	0.85 (0.70, 1.02), 0.16	0.98 (0.81, 1.18), 0.07	·	✓	✗	✗

Note

Empty cells indicate nonsignificant relationships; all other relationships are significant at *p* < 0.05. Bold numbers indicate slopes with significant deviations from isometry (H0: slope = 1). Ticks in right columns indicate accordance with isometric and anisometric patterns described by Kerkhoff et al. (2006) for the specific combination of plant organs, crosses indicate no accordance.

**Figure 4:**
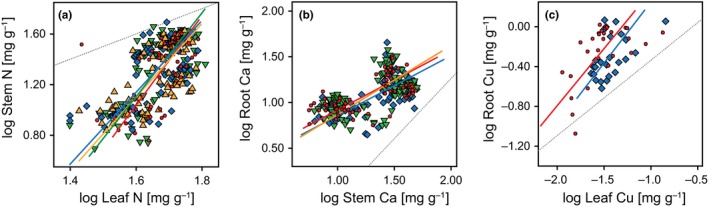
Standardized major axis regression (SMA) relationships of N (a), Ca (b), and Cu (c) between stems, leaves, and roots. Shown are values for control (red circles, red line), penicillin (yellow triangles, yellow line), sulfadiazine (green triangles, green line), and tetracycline (blue diamonds, blue line). All relationships are significant at *p* < 0.05. The 1:1 line (gray) is shown in each graph, indicating the course of isometric slope

Among treatments, expected patterns were mostly found in control treatments (14 of 24 tested cases, indicated by ticks in Table 2), but less so in the antibiotic treatments (10 of 22 for tetracycline, 7 of 21 for sulfadiazine, and 6 of 25 for penicillin, see also examples in Figure 4).

## 4 DISCUSSION

To our knowledge, this study is the first to demonstrate effects of in vivo concentrations of antibiotics on plant organ element contents. While most other studies on antibiotic‐effects on plants used much higher concentrations (Hillis, Fletcher, Solomon, & Sibley, 2011; Jin, Chen, Sun, Zhou, & Liu, 2009; Li et al., 2011; Pan & Chu, 2016; Yang et al., 2010; Ziolkowska, Piotrowicz‐Cieslak, Margas, Adomas, & Nalecz‐Jawecki, 2015), we used comparatively low concentrations, which were also detected in agricultural soils (Thiele‐Bruhn, 2003). Our results show that antibiotics, even in these low concentrations, affect plant element content, with similar effects expected for real‐world scenarios. We show significant species‐specific responses as well as organ‐specific responses to different antibiotics, with roots responding most strongly.

Our analyses showed that all elements responded to the antibiotic treatments, but with different response directions. Some element contents were lower in the antibiotic than in the control treatments, while the reverse was found for other elements. In general, plant element contents were most responsive to penicillin, followed by sulfadiazine and tetracycline, which was also found for other traits in the same four species and experimental setup (i.e., biomass allocation, growth rates, chlorophyll content and others, see Minden et al., 2017).

Moreover, responses to antibiotics differed between plant species: element contents in the two herb species *B. napus* and *C. bursa‐pastoris* were mostly reduced by antibiotics, whereas contents were increased in the two grass species *T. aestivum* and *A. spica‐venti*. Species‐specific results have been shown by other studies on antibiotic‐induced responses, for example, on germination (Liu et al., 2009; Minden et al., 2017), postgerminative development (Migliore et al., 1997), root elongation (Pan & Chu, 2016), root and shoot lengths (Hillis et al., 2011), or bioaccumulation (Migliore, Brambilla, Cozzolino, & Gaudio, 1995).

It is interesting that organ‐specific responses were unrelated to species, family, or functional group. Roots were, in general, most strongly affected by the treatments and stems were least affected. To date, there are only few other experimental studies, with which our results can be directly compared. Michelini et al. (2012) studied the effects of sulfadiazine on *Salix fragilis* and *Zea mays* plants and found disequilibria in C, N, K, and Ca concentrations for the two species. However, they did not detect the pronounced organ‐specific responses found in our study. Further, they used concentrations of 10 and 200 mg/kg soil, whereas our maximum concentration was 10 μg/L solution (or 0.38 μg/kg soil), which makes their results only partly comparable to ours. Moreover, our results on organ‐specific responses of plant elements to antibiotics are in line with other studies showing that roots are generally most affected by antibiotic treatments. For example, Pierattini et al. (2016) showed that erythromycin concentrations were tenfold higher in roots than in aerial plant parts in *Populus alba* (see also Pan, Wong, & Chu, 2014). In *Lythrum salicaria*, Migliore et al. (2010) detected toxic effects of sulfadimethoxine on roots, cotyledons, and cotyledons petioles for all applied concentrations, while internodes and leaf lengths showed dose‐dependent responses. Other root‐related effects reported for antibiotics were decreased root length, root elongation, and numbers of lateral roots, indicating possible consequences for plant water uptake (Michelini et al., 2012; Piotrowicz‐Cieslak, Adomas, Nalecz‐Jawecki, & Michalczyk, 2010).

Plants regulate the uptake rates of elements to ensure element homeostasis (i.e., maintenance of constant body concentrations despite fluctuations in environmental resources, Bradshaw, Kautsky, & Kumblad, 2012; Cannon, 1929). Homeostatic regulation is needed to maintain element contents above certain thresholds under which growth is impaired, whereas excessive uptakes of elements can cause toxic effects (Güsewell, 2004). Antibiotics, once taken up by the root, may be transported to the aerial tissues (i.e., leaves, stems, flowers, fruits) of plants (Briggs, Bromilow, & Evans, 1982; Trapp & Mc Farlane, 1995), and may be stored, transformed or sequestered by plant metabolic processes (“green liver model,” Burken, 2003; Sandermann, 2004). Our results suggest several possible ways of antibiotic‐induced effects on element contents in plants: (a) Uptake of elements through the root may be impacted and hence decreases plant element contents, especially in roots, which may explain the low root P contents in plants treated with antibiotics. (b) Only in a few cases, nutrient contents were higher in antibiotic‐treated plants, like K content in stems of *T. aestivum*, which may result from an antibiotic‐induced disrupted distribution of elements across the plant. More research clearly is needed to better understand how antibiotics may affect nutrient uptake and distribution of nutrients across plants. Note that in most of the combinations tested, especially for leaves and stems, we did not detect significant differences between control and antibiotic treatments, which may result from the deliberately chosen low antibiotic soil concentrations, but all elements responded significantly to at least one antibiotic treatment. Furthermore and as a clear limitation to our study, the role of microorganisms on the element contents of the tested plant species were not taken into account. It was shown that soil bacteria were significantly affected by antibiotics (Thiele‐Bruhn & Beck, 2005; Wei, Wu, Nie, Yediler, & Wong, 2009; Yang, Zhang, Zhu, & Zhang, 2009). Obviously, a critical future question is to what extent our results were driven by antibiotics alone or by a combination of antibiotics and (impeded) microbial activity. As such, the results of the present study only reflect the responses of plant element contents to the antibiotic treatments, while we cannot make a distinction into direct (uptake and metabolization of the compound by the plant) and indirect (through microbial activity) effects of antibiotics.

Kerkhoff et al. (2006) found patterns of isometric and anisometric scaling relationships between leaves, stems, and roots (and reproductive structures). The results of our pooled dataset of all four species supported their findings for stems versus leaves (*Y* vs. *X*). However, for the remaining combinations (roots vs. stems and root vs. leaves), their patterns could only partly be reproduced. Moreover, expected isometric and anisometric scaling relationships were most often found for the control treatments, but less so for the antibiotic treatments, with penicillin yielding the lowest number of concordant patterns. This is well in line with our results revealing strongest effects on tissue element concentrations for penicillin and weakest effects for tetracycline.

A similar analysis on how stressors may affect scaling relationships, yet with field data from salt marshes, has been conducted by Minden and Kleyer (2014). They concluded that “structural” organs, like stems and roots, were less homeostatic (i.e., they exerted high elemental fluctuations due to environmental constraints) than “metabolic” organs like leaves and diaspores. However, they identified stems as the organs with lowest homeostasis in response to nutrient availability and salt stress. Our results indicate that under antibiotic stress, roots exhibit a lower homeostasis than under natural conditions. A low homeostatic element composition in one organ also implies effects on other plant organs, because the overall performance of a plant depends on the interplay between its organs and their specific functions (see Kleyer & Minden, 2015). In a consequent manner, low homeostatic responses in one or more plant organs may affect the performance of the whole plant, which may further scale up to the community level with consequences for community composition and other trophic levels.

In summary, the results from our study show that antibiotics in concentrations as found in agricultural landscapes can affect element contents of plants, particularly in roots. In roots, they may lead to low‐element homeostasis, altering the scaling between roots and other plant organs, which may affect metabolic processes and ultimately the performance of a plant.

## CONFLICT OF INTEREST

None declared.

## AUTHOR CONTRIBUTIONS

VM, GP, and SDL designed research; VM performed research; VM and BS analyzed samples; all authors contributed critically to the draft and gave final approval for publication.
