# Supplementary material for: Antibiotic‐induced effects on scaling relationships and on plant element contents in herbs and grasses
Source: Ecol Evol. 2018 Jun 2;8(13):6699–713. doi: 10.1002/ece3.4168 (PMC6053569; doi:10.1002/ece3.4168)
Supplement: Supplementary file 1 [file ECE3-8-6699-s001.docx]

**APPENDIX**

**Appendix 1** Primary functions of plant elements and their chemical forms available to plants. List is not exhaustive, for more information see Marschner (2012) and Sterner and Elser (2002).

| Element | Chemical form | Function in plants |
| --- | --- | --- |
| N | NO_2_^-^, NO_3_^-^ / NH_4_^+^ | Proteins, nucleic acids, chlorophyll, hormones, secondary metabolites |
| P | H_2_PO_4_^-^ / HPO_4_^2-^ | Nucleic acids, ATP, phospholipids, RUBSICO |
| K | K^+^ | Activation of enzymes, osmoregulation, loading of sucrose |
| C | CO_2_, HCO_3_^-^ | Carbohydrates, organic acids, enzymes, hormones |
| Ca | Ca^2+^ | Secondary messenger, cell wall, membrane stabilisation, osmoregulation |
| S | SO_4_^2-^ | Proteins, amino acids |
| Cu | Cu^2+^ | Electron transport, activation of enzymes |
| Mg | Mg^2+^ | Chlorophyll, protein synthesis |
| Fe | Fe^2+^ / Fe^3+^ | Synthesis of chlorophyll, electron transfer in photosynthesis and respiration |
| Mn | Mn^2+^ | Activation of enzymes, photosynthetic water splitting |
| Na | Na^+^ | Partially as replacement for K, osmoregulation |

**Appendix 2:** Summary of reduced major axis (SMA) regression results for all combinations of plant organs for each element for the plant species-specific dataset. Given are slopes of the regression and 95% confidence intervals in parentheses, and correlation coefficients (r) for *Brassica napus* (left column), *Capsella bursa-pastoris*, *Triticum aestivum* and *Apera spica-venti* (right column). Treatments are control (C), penicillin (P), sulfadiazine (S) and tetracycline (T). NS indicates a non-significant relationship; all other relationships are significant at P < 0.05. Bold numbers indicate slopes with significant deviations from isometry (H0: slope = 1). Right column indicates underlying hypotheses regarding slope following Kerkhoff *et al.* (2006).

| SMA of Nitrogen | | | | *B. napus* | *C. bursa-pastoris* | *T. aestivum* | *A. spica-venti* | Hypothesis |
| --- | --- | --- | --- | --- | --- | --- | --- | --- |
| stem | | leaf | C | **1.86 (1.21, 2.86), 0.19** | -0.78 (-1.50, -0.41), 0.52 | NS | NS | Slope > 1 (H1) |
|  | |  | P | NS | NS | 1.08 (0.73, 1.58), 0.36 | **1.49 (0.99, 2.25), 0.18** |  |
|  | |  | S | **2.06 (1.45, 2.92), 0.17** | NS | **1.57 (1.21, 2.04), 0.58** | **1.51 (1.02, 2.21), 0.29** |  |
|  | |  | T | 1.12 (0.79, 1.58), 0.17 | NS | 1.26 (0.89, 1.78), 0.37 | NS |  |
| root | | stem | C | NS | NS | NS | 1.20 (0.78, 1.86), 0.49 | Slope ~ 1 (H0) |
|  | |  | P | NS | **0.59 (0.39, 0.90), 0.25** | NS | NS |  |
|  | |  | S | **0.49 (0.35, 0.68), 0.26** | NS | -1.00 (-1.43, -0.70), 0.21 | NS |  |
|  | |  | T | 0.77 (0.55, 1.08), 0.22 | NS | NS | NS |  |
| root | | leaf | C | NS | NS | **-1.82 (-2.88, -1.15), 0.32** | NS | Slope < 1 (H1) |
|  | |  | P | NS | NS | NS | NS |  |
|  | |  | S | NS | NS | **-1.45 (-2.09, -1.01), 0.25** | NS |  |
|  | |  | T | NS | NS | NS | NS |  |
|  | |  |  |  |  |  |  |  |
| SMA of Phosphorus | | | | *B. napus* | *C. bursa-pastoris* | *T. aestivum* | *A. spica-venti* |  |
| stem | | leaf | C | **1.65 (1.09, 2.51), 0.24** | -1.18 (-2.05, -0.67), 0.57 | 0.80 (0.59, 1.09), 0.68 | NS | Slope > 1 |
|  | |  | P | 1.44 (0.96, 2.15), 0.21 | NS | 0.94 (0.67, 1.33), 0.41 | 1.01 (0.69, 1.46), 0.22 |  |
|  | |  | S | **1.62 (1.13, 2.33), 0.08** | NS | 0.95 (0.76, 1.19), 0.68 | 0.78 (0.56, 1.08), 0.50 |  |
|  | |  | T | 1.29 (0.93, 1.79), 0.26 | 1.05 (0.71, 1.54), 0.14 | **1.49 (1.10, 2.03), 0.42** | **0.57 (0.42, 0.77), 0.42** |  |
| root | | stem | C | NS | NS | 0.92 (0.61, 1.38), 0.39 | NS | Slope ~ 1 |
|  | |  | P | NS | NS | 0.76 (0.57, 1.01), 0.52 | NS |  |
|  | |  | S | NS | NS | 0.82 (0.64, 1.05), 0.61 | NS |  |
|  | |  | T | **0.68 (0.48, 0.95), 0.19** | 0.93 (0.64, 1.34), 0.24 | 1.04 (0.72, 1.51), 0.17 | NS |  |
| root | | leaf | C | **-2.31 (-3.47, -1.54), 0.29** | 0.89 (0.71, 1.12), 0.87 | 0.73 (0.50, 1.06), 0.56 | NS | Slope < 1 |
|  | |  | P | NS | NS | 0.80 (0.60, 1.08), 0.52 | NS |  |
|  | |  | S | NS | 1.02 (0.75, 1.39), 0.38 | 0.79 (0.58, 1.07), 0.43 | NS |  |
|  | |  | T | 0.87 (0.62, 1.23), 0.18 | 0.99 (0.70, 1.40), 0.27 | **1.57 (1.09, 2.25), 0.18** | NS |  |
|  | |  |  |  |  |  |  |  |
| SMA of Potassium | | | | *B. napus* | *C. bursa-pastoris* | *T. aestivum* | *A. spica-venti* |  |
| stem | | leaf | C | NS | NS | **-3.63 (-5.63, -2.34), 0.37** | **2.56 (1.67, 3.92), 0.35** | Slope > 1 |
|  | |  | P | NS | **2.26 (1.59, 3.21), 0.16** | NS | NS |  |
|  | |  | S | NS | NS | NS | NS |  |
|  | |  | T | NS | **1.80 (1.28, 2.52), 0.36** | NS | NS |  |
| root | | stem | C | NS | NS | **0.65 (0.45, 0.95), 0.51** | 1.51 (0.93, 2.45), 0.23 | Slope ~ 1 |
|  | |  | P | NS | NS | NS | NS |  |
|  | |  | S | NS | NS | NS | NS |  |
|  | |  | T | NS | **2.70 (1.85, 3.94), 0.23** | **2.85 (1.94, 4.18), 0.09** | NS |  |
| root | | leaf | C | NS | NS | **-2.36 (-3.77, -1.48), 0.29** | NS | Slope < 1 |
|  | |  | P | NS | NS | NS | NS |  |
|  | |  | S | NS | NS | **4.88 (3.49, 6.84), 0.30** | NS |  |
|  | |  | T | NS | NS | NS | NS |  |
|  | |  |  |  |  |  |  |  |
| SMA of Carbon | | | | *B. napus* | *C. bursa-pastoris* | *T. aestivum* | *A. spica-venti* |  |
| stem | | leaf | C | **1.40 (1.04, 1.89), 0.62** | NS | 0.85 (0.54, 1.35), 0.25 | NS | Slope > 1 |
|  | |  | P | NS | NS | NS | NS |  |
|  | |  | S | 1.28 (0.92, 1.80), 0.24 | NS | 1.01 (0.78, 1.31), 0.59 | NS |  |
|  | |  | T | 1.28 (0.91, 1.79), 0.22 | NS | 0.82 (0.59, 1.15), 0.42 | 1.25 (0.86, 1.8), 0.20 |  |
| root | | stem | C | NS | NS | NS | NS | Slope ~ 1 |
|  | |  | P | NS | NS | NS | NS |  |
|  | |  | S | NS | NS | NS | NS |  |
|  | |  | T | NS | NS | NS | NS |  |
| root | | leaf | C | NS | NS | **-0.23 (-0.37, -0.15), 0.32** | NS | Slope < 1 |
|  | |  | P | NS | NS | NS | NS |  |
|  | |  | S | NS | NS | NS | NS |  |
|  | |  | T | NS | NS | NS | NS |  |
|  | |  |  |  |  |  |  |  |
|  | |  |  |  |  |  |  |  |
|  | |  |  |  |  |  |  |  |
|  | |  |  |  |  |  |  |  |
| *Appendix 2 continued* | | | | | | | | |
|  | | | | | | | | |
| SMA of Calcium | | | | *B. napus* | *C. bursa-pastoris* | *T. aestivum* | *A. spica-venti* |  |
| stem | | leaf | C | NS | NS | 1.11 (0.71, 1.74), 0.35 | NS | Slope > 1 |
|  | |  | P | NS | 2.26 (1.7, 3.01), 0.49 | 1.06 (0.76, 1.48), 0.38 | NS |  |
|  | |  | S | NS | 2.22 (1.64, 3.00), 0.41 | **1.32 (0.98, 1.78), 0.46** | **1.68 (1.19, 2.37), 0.44** |  |
|  | |  | T | NS | NS | **1.37 (1.08, 1.73), 0.69** | **1.43 (1.07, 1.91), 0.52** |  |
| root | | stem | C | NS | NS | NS | NS | Slope ~ 1 |
|  | |  | P | NS | NS | NS | NS |  |
|  | |  | S | NS | NS | NS | NS |  |
|  | |  | T | **1.84 (1.30, 2.62), 0.14** | NS | NS | NS |  |
| root | | leaf | C | NS | NS | NS | NS | Slope < 1 |
|  | |  | P | -1.78 (-2.53, -1.26), 0.16 | NS | **-0.50 (-0.72, -0.34), 0.18** | NS |  |
|  | |  | S | NS | NS | **-0.44 (-0.63, -0.31), 0.24** | NS |  |
|  | |  | T | NS | NS | NS | NS |  |
|  | |  |  |  |  |  |  |  |
| SMA of Sulfur | | | | *B. napus* | *C. bursa-pastoris* | *T. aestivum* | *A. spica-venti* |  |
| stem | leaf | | C | **1.82 (1.33, 2.51), 0.57** | NS | NS | NS | Slope > 1 |
|  | | | P | **1.56 (1.07, 2.27), 0.32** | NS | NS | NS |  |
|  | | | S | 1.33 (0.96, 1.86), 0.23 | NS | NS | NS |  |
|  | | | T | 1.36 (0.98, 1.87), 0.28 | 1.27 (0.87, 1.86), 0.19 | **4.87 (3.31, 7.16), 0.15** | NS |  |
| root | stem | | C | NS | NS | **-0.55 (-0.82, -0.36), 0.39** | NS | Slope ~ 1 |
|  | | | P | -0.70 (-1.03, -0.47), 0.25 | NS | -1.06 (-1.61, -0.70), 0.22 | NS |  |
|  | | | S | NS | NS | **-0.58 (-0.82, -0.40), 0.19** | NS |  |
|  | | | T | NS | NS | NS | NS |  |
| root | leaf | | C | NS | NS | NS | NS | Slope < 1 |
|  | | | P | -1.00 (-1.4, -0.72), 0.22 | NS | NS | NS |  |
|  | | | S | NS | **-1.65 (-2.40, -1.14), 0.15** | NS | NS |  |
|  | | | T | NS | NS | NS | NS |  |
|  | |  |  |  |  |  |  |  |
| SMA of Copper | | | | *B. napus* | *C. bursa-pastoris* | *T. aestivum* | *A. spica-venti* |  |
| stem | | leaf | C | **0.57 (0.38, 0.86), 0.36** | NS | **2.95 (1.81, 4.81), 0.48** | NS | Slope > 1 |
|  | |  | P | 1.08 (0.74, 1.58), 0.31 | **1.68 (1.20, 2.34), 0.28** | NS | NS |  |
|  | |  | S | 0.79 (0.56, 1.12), 0.19 | 1.19 (0.86, 1.64), 0.34 | **3.44 (2.33, 5.08), 0.47** | NS |  |
|  | |  | T | NS | **1.84 (1.26, 2.67), 0.22** | NS | NS |  |
| root | | stem | C | NS | NS | 1.06 (0.69, 1.64), 0.49 | NS | Slope ~ 1 |
|  | |  | P | NS | **8.40 (4.65, 15.19), 0.60** | 1.04 (0.76, 1.43), 0.47 | 1.15 (0.78, 1.71), 0.42 |  |
|  | |  | S | NS | 1.20 (0.87, 1.67), 0.37 | NS | NS |  |
|  | |  | T | 0.75 (0.53, 1.07), 0.14 | NS | 1.11 (0.71, 1.75), 0.33 | NS |  |
| root | | leaf | C | NS | NS | NS | NS | Slope < 1 |
|  | |  | P | NS | **1.74 (1.26, 2.40), 0.31** | **1.81 (1.25, 2.62), 0.22** | NS |  |
|  | |  | S | NS | **1.53 (1.17, 2.01), 0.55** | **4.15 (2.73, 6.31), 0.19** | NS |  |
|  | |  | T | NS | **1.72 (1.19, 2.49), 0.15** | **2.74 (1.80, 4.16), 0.19** | NS |  |
|  | |  |  |  |  |  |  |  |
| SMA of Magnesium | | | | *B. napus* | *C. bursa-pastoris* | *T. aestivum* | *A. spica-venti* |  |
| stem | | leaf | C | NS | NS | NS | NS | Slope > 1 |
|  | |  | P | NS | NS | NS | NS |  |
|  | |  | S | NS | NS | 0.75 (0.56, 1.01), 0.47 | NS |  |
|  | |  | T | NS | NS | 0.92 (0.67, 1.26), 0.45 | 0.94 (0.7, 1.26), 0.49 |  |
| root | | stem | C | NS | NS | NS | 1.12 (0.71, 1.78), 0.31 | Slope ~ 1 |
|  | |  | P | NS | NS | NS | NS |  |
|  | |  | S | NS | NS | **-0.76 (-0.94, -0.61), 0.69** | NS |  |
|  | |  | T | 0.89 (0.63, 1.24), 0.21 | NS | NS | NS |  |
| root | | leaf | C | NS | NS | **-0.59 (-0.83, -0.42), 0.62** | NS | Slope < 1 |
|  | |  | P | **-1.38 (-1.90, -1.00), 0.28** | NS | **-0.61 (-0.86, -0.43), 0.34** | NS |  |
|  | |  | S | NS | **-1.92 (-2.77, -1.33), 0.17** | **-0.61 (-0.80, -0.46), 0.52** | NS |  |
|  | |  | T | **1.51 (1.07, 2.13), 0.18** | NS | -0.73 (-1.00, -0.53), 0.44 | NS |  |
|  | |  |  |  |  |  |  |  |
| SMA of Iron | | | | *B. napus* | *C. bursa-pastoris* | *T. aestivum* | *A. spica-venti* |  |
| stem | | leaf | C | NS | NS | NS | NS | Slope > 1 |
|  | |  | P | NS | NS | NS | NS |  |
|  | |  | S | NS | **1.52 (1.06, 2.18), 0.20** | **1.60 (1.12, 2.30), 0.17** | NS |  |
|  | |  | T | NS | NS | NS | NS |  |
| root | | stem | C | NS | -1.00 (-1.70, -0.58), 0.68 | NS | NS | Slope ~ 1 |
|  | |  | P | NS | NS | **0.67 (0.46, 0.99), 0.16** | NS |  |
|  | |  | S | NS | NS | NS | NS |  |
|  | |  | T | NS | **0.47 (0.32, 0.68), 0.26** | NS | NS |  |
| root | | leaf | C | NS | 0.79 (0.50, 1.27), 0.34 | NS | NS | Slope < 1 |
|  | |  | P | NS | **0.69 (0.48, 0.99), 0.13** | **0.35 (0.26, 0.47), 0.48** | NS |  |
|  | |  | S | NS | NS | NS | NS |  |
|  | |  | T | NS | NS | NS | NS |  |
|  | |  |  |  |  |  |  |  |
|  | |  |  |  |  |  |  |  |
|  | |  |  |  |  |  |  |  |
|  | |  |  |  |  |  |  |  |
| *Appendix 2 continued* | | | | | | | | |
|  | | | | | | | | |
| SMA of Manganese | | | | *B. napus* | *C. bursa-pastoris* | *T. aestivum* | *A. spica-venti* |  |
| stem | | leaf | C | **0.57 (0.38, 0.86), 0.36** | NS | NS | 0.84 (0.53, 1.32), 0.27 | Slope > 1 |
|  | |  | P | 1.08 (0.74, 1.58), 0.31 | **1.68 (1.20, 2.34), 0.28** | 1.03 (0.69, 1.53), 0.24 | NS |  |
|  | |  | S | 0.79 (0.56, 1.12), 0.19 | 1.19 (0.86, 1.64), 0.34 | 0.82 (0.56, 1.19), 0.18 | 0.87 (0.58, 1.29), 0.20 |  |
|  | |  | T | NS | **1.84 (1.26, 2.67), 0.22** | **1.47 (1.09, 1.99), 0.48** | NS |  |
| root | | stem | C | NS | NS | NS | NS | Slope ~ 1 |
|  | |  | P | NS | 1.00 (0.72, 1.37), 0.39 | 1.28 (0.86, 1.90), 0.17 | NS |  |
|  | |  | S | NS | 1.20 (0.87, 1.67), 0.37 | 1.07 (0.79, 1.44), 0.42 | NS |  |
|  | |  | T | 0.75 (0.53, 1.07), 0.14 | NS | **1.53 (1.11, 2.12), 0.35** | NS |  |
| root | | leaf | C | 1.00 (0.67, 1.48), 0.34 | NS | **1.53 (1.01, 2.30), 0.46** | NS | Slope < 1 |
|  | |  | P | NS | **1.74 (1.26, 2.40), 0.31** | NS | NS |  |
|  | |  | S | NS | **1.53 (1.17, 2.01), 0.55** | 0.92 (0.64, 1.32), 0.25 | NS |  |
|  | |  | T | NS | **1.72 (1.19, 2.49), 0.15** | **2.26 (1.55, 3.29), 0.17** | NS |  |
|  | |  |  |  |  |  |  |  |
| SMA of Sodium | | | | *B. napus* | *C. bursa-pastoris* | *T. aestivum* | *A. spica-venti* |  |
| stem | | leaf | C | 1.34 (0.92, 1.94), 0.40 | NS | **0.60 (0.38, 0.94), 0.30** | 0.98 (0.65, 1.49), 0.40 | Slope > 1 |
|  | |  | P | 0.80 (0.60, 1.07), 0.61 | **1.58 (1.09, 2.29), 0.16** | 0.73 (0.53, 1.00), 0.50 | 1.07 (0.74, 1.56), 0.26 |  |
|  | |  | S | **0.75 (0.64, 0.89), 0.81** | 1.28 (0.88, 1.86), 0.10 | **0.60 (0.47, 0.75), 0.67** | 1.00 (0.71, 1.40), 0.45 |  |
|  | |  | T | 0.78 (0.61, 1.00), 0.59 | **1.53 (1.16, 2.01), 0.58** | **0.63 (0.48, 0.83), 0.58** | 1.07 (0.86, 1.33), 0.73 |  |
| root | | stem | C | NS | NS | NS | NS | Slope ~ 1 |
|  | |  | P | NS | NS | NS | -1.11 (-1.70, -0.73), 0.34 |  |
|  | |  | S | NS | NS | NS | NS |  |
|  | |  | T | 0.81 (0.58, 1.12), 0.26 | NS | **0.65 (0.46, 0.92), 0.28** | NS |  |
| root | | leaf | C | NS | **0.36 (0.23, 0.57), 0.46** | **-0.30 (-0.47, -0.19), 0.38** | NS | Slope < 1 |
|  | |  | P | **0.50 (0.35, 0.72), 0.13** | **0.69 (0.49, 0.99), 0.16** | NS | NS |  |
|  | |  | S | NS | NS | NS | NS |  |
|  | |  | T | NS | NS | **0.42 (0.29, 0.62), 0.23** | NS |  |

**Appendix 3:** Means and standard deviations of each element concentration (all in mg/g) in leaves, stems and roots for *Brassica napus*, *Capsella bursa-pastoris*, *Triticum aestivum* and *Apera spica-venti*. Treatments are control, penicillin (1, 5, 10 µg/L), sulfadiazine (1, 5, 10 µg/L) and tetracycline (1, 5, 10 µg/L).

| **Nitrogen** | ***B. napus*** | ***C. bursa-past.*** | ***T. aestivum*** | ***A. spica-venti*** | ***B. napus*** | ***C. bursa-past.*** | ***T. aestivum*** | ***A. spica-venti*** | ***B. napus*** | ***C. bursa-past.*** | ***T. aestivum*** | ***A. spica-venti*** |
| --- | --- | --- | --- | --- | --- | --- | --- | --- | --- | --- | --- | --- |
|  | Leaves | Leaves | Leaves | Leaves | Stems | Stems | Stems | Stems | Roots | Roots | Roots | Roots |
| **C** | 41.66 ± 5.9 | 50.75 ± 9.0 | 49.01 ± 4.7 | 53.88 ± 3.0 | 9.86 ± 2.7 | 22.92 ± 4.8 | 30.63 ± 5.4 | 35.04 ± 4.0 | 16.57 ± 2.8 | 24.62 ± 2.3 | 17.16 ± 3.1 | 18.76 ± 2.5 |
| **P1** | 36.76 ± 4.5 | 53.20 ± 4.3 | 50.29 ± 7.1 | 54.21 ± 2.4 | 9.48 ± 1.8 | 19.72 ± 3.8 | 32.92 ± 3.1 | 37.92 ± 5.2 | 15.60 ± 2.7 | 24.61 ± 2.8 | 14.06 ± 3.9 | 19.11 ± 2.9 |
| **P5** | 37.82 ± 7.1 | 51.32 ± 4.7 | 52.15 ± 4.1 | 49.81 ± 5.6 | 10.92 ± 1.2 | 16.62 ± 2.9 | 32.87 ± 4.3 | 33.39 ± 1.5 | 16.31 ± 1.9 | 23.46 ± 2.6 | 8.96 ± 4.0 | 16.85 ± 1.1 |
| **P10** | 39.07 ± 4.8 | 30.41 ± 15.5 | 52.87 ± 4.5 | 48.86 ± 4.7 | 11.26 ± 2.6 | 15.61 ± 1.4 | 33.99 ± 4.4 | 30.52 ± 6.7 | 15.80 ± 1.0 | 22.43 ± 2.0 | 11.40 ± 3.3 | 20.03 ± 1.2 |
| **S1** | 40.27 ± 5.6 | 46.03 ± 7.5 | 50.26 ± 8.1 | 53.92 ± 1.6 | 9.45 ± 4.7 | 18.60 ± 2.1 | 34.41 ± 5.7 | 37.45 ± 2.7 | 15.45 ± 2.2 | 24.12 ± 1.9 | 14.27 ± 1.9 | 20.01 ± 2.7 |
| **S5** | 35.92 ± 6.3 | 51.71 ± 8.7 | 46.79 ± 4.4 | 52.04 ± 4.9 | 8.77 ± 2.0 | 21.20 ± 2.4 | 33.71 ± 6.7 | 37.52 ± 4.4 | 17.26 ± 2.9 | 25.02 ± 2.3 | 15.17 ± 3.2 | 18.39 ± 2.6 |
| **S10** | 39.67 ± 5.0 | 56.16 ± 8.8 | 47.42 ± 4.6 | 52.15 ± 5.0 | 10.81 ± 1.4 | 26.14 ± 8.3 | 34.27 ± 5.8 | 36.13 ± 4.4 | 18.03 ± 1.8 | 25.11 ± 2.4 | 16.43 ± 2.7 | 18.40 ± 1.3 |
| **T1** | 36.71 ± 7.3 | 52.61 ± 5.1 | 43.24 ± 2.3 | 51.27 ± 3.9 | 10.76 ± 3.4 | 21.63 ± 3.8 | 32.53 ± 5.1 | 35.10 ± 3.2 | 17.00 ± 2.5 | 23.54 ± 2.6 | 14.84 ± 2.9 | 17.10 ± 2.4 |
| **T5** | 36.10 ± 6.9 | 52.43 ± 6.2 | 46.28 ± 6.1 | 52.06 ± 2.0 | 8.82 ± 1.1 | 19.83 ± 1.7 | 29.67 ± 3.4 | 38.47 ± 5.3 | 15.08 ± 2.4 | 20.55 ± 1.9 | 16.99 ± 1.8 | 15.70 ± 2.7 |
| **T10** | 37.65 ± 3.8 | 51.26 ± 5.6 | 45.94 ± 6.2 | 54.59 ± 3.6 | 9.53 ± 0.8 | 18.84 ± 4.4 | 32.46 ± 5.4 | 36.79 ± 2.7 | 15.80 ± 1.8 | 21.61 ± 2.4 | 17.67 ± 2.6 | 17.17 ± 2.8 |
| **Phosphorus** | ***B. napus*** | ***C. bursa-past.*** | ***T. aestivum*** | ***A. spica-venti*** | ***B. napus*** | ***C. bursa-past.*** | ***T. aestivum*** | ***A. spica-venti*** | ***B. napus*** | ***C. bursa-past.*** | ***T. aestivum*** | ***A. spica-venti*** |
|  | Leaves | Leaves | Leaves | Leaves | Stems | Stems | Stems | Stems | Roots | Roots | Roots | Roots |
| **C** | 3.59 ± 0.6 | 4.66 ± 1.7 | 4.90 ± 1.6 | 4.08 ± 0.5 | 1.61 ± 0.4 | 2.10 ± 0.6 | 4.13 ± 1.0 | 4.74 ± 0.6 | 2.45 ± 1.7 | 3.97 ± 1.5 | 3.88 ± 0.9 | 5.53 ± 0.9 |
| **P1** | 3.80 ± 0.5 | 3.53 ± 0.6 | 5.88 ± 2.0 | 5.49 ± 1.5 | 2.05 ± 0.8 | 1.81 ± 0.3 | 5.58 ± 1.2 | 5.38 ± 0.6 | 2.19 ± 0.4 | 2.67 ± 0.4 | 4.29 ± 1.1 | 6.86 ± 1.1 |
| **P5** | 3.45 ± 0.9 | 3.39 ± 0.2 | 6.23 ± 1.5 | 4.65 ± 1.1 | 1.78 ± 0.4 | 1.86 ± 0.3 | 6.77 ± 1.6 | 4.86 ± 0.8 | 2.09 ± 0.2 | 2.62 ± 0.4 | 4.86 ± 0.7 | 5.50 ± 1.5 |
| **P10** | 3.61 ± 0.4 | 3.52 ± 0.7 | 5.85 ± 1.5 | 4.94 ± 1.1 | 2.21 ± 0.6 | 1.79 ± 0.4 | 5.18 ± 1.0 | 4.18 ± 1.4 | 2.09 ± 0.2 | 2.64 ± 0.4 | 4.45 ± 0.8 | 5.40 ± 1.7 |
| **S1** | 3.57 ± 0.5 | 3.79 ± 0.9 | 4.76 ± 2.0 | 4.90 ± 1.2 | 1.87 ± 0.6 | 1.81 ± 0.5 | 4.63 ± 1.2 | 4.97 ± 0.9 | 2.10 ± 0.4 | 2.71 ± 0.5 | 3.82 ± 0.6 | 5.67 ± 0.5 |
| **S5** | 3.40 ± 0.5 | 3.61 ± 0.6 | 4.63 ± 1.0 | 4.96 ± 0.8 | 2.05 ± 0.3 | 2.05 ± 0.4 | 4.31 ± 1.2 | 5.39 ± 0.7 | 2.38 ± 0.3 | 2.69 ± 0.5 | 4.15 ± 1.3 | 5.60 ± 0.7 |
| **S10** | 3.22 ± 0.4 | 4.12 ± 0.9 | 4.56 ± 1.3 | 4.76 ± 1.6 | 1.56 ± 0.2 | 2.90 ± 1.4 | 4.65 ± 1.3 | 4.79 ± 1.1 | 2.13 ± 0.2 | 3.20 ± 0.7 | 3.70 ± 0.9 | 5.94 ± 0.7 |
| **T1** | 3.10 ± 0.6 | 3.51 ± 0.4 | 4.90 ± 0.5 | 3.68 ± 0.8 | 1.65 ± 0.6 | 1.87 ± 0.4 | 4.54 ± 1.1 | 4.36 ± 1.0 | 2.36 ± 0.7 | 3.16 ± 0.3 | 4.11 ± 1.3 | 4.40 ± 0.7 |
| **T5** | 3.76 ± 1.3 | 4.02 ± 0.6 | 5.21 ± 1.4 | 4.57 ± 0.7 | 1.85 ± 0.5 | 1.86 ± 0.5 | 4.86 ± 1.0 | 5.10 ± 0.6 | 2.24 ± 0.3 | 2.95 ± 0.6 | 5.17 ± 2.4 | 4.47 ± 0.8 |
| **T10** | 3.37 ± 0.4 | 3.85 ± 1.2 | 4.47 ± 1.1 | 4.89 ± 1.4 | 1.70 ± 0.3 | 1.97 ± 0.4 | 4.16 ± 1.3 | 5.32 ± 0.5 | 1.93 ± 0.2 | 2.90 ± 0.8 | 4.19 ± 1.0 | 4.54 ± 0.7 |
| **Potassium** | ***B. napus*** | ***C. bursa-past.*** | ***T. aestivum*** | ***A. spica-venti*** | ***B. napus*** | ***C. bursa-past.*** | ***T. aestivum*** | ***A. spica-venti*** | ***B. napus*** | ***C. bursa-past.*** | ***T. aestivum*** | ***A. spica-venti*** |
|  | Leaves | Leaves | Leaves | Leaves | Stems | Stems | Stems | Stems | Roots | Roots | Roots | Roots |
| **C** | 36.66 ± 4.3 | 45.28 ± 8.8 | 57.97 ± 7.0 | 52.88 ± 7.0 | 22.16 ± 5.5 | 34.66 ± 5.0 | 39.44 ± 12.1 | 28.93 ± 9.4 | 4.55 ± 2.1 | 2.53 ± 1.6 | 1.89 ± 0.5 | 1.66 ± 0.9 |
| **P1** | 31.55 ± 4.3 | 51.72 ± 9.2 | 58.87 ± 4.3 | 51.60 ± 5.1 | 24.67 ± 7.2 | 32.74 ± 7.0 | 51.88 ± 4.3 | 32.70 ± 10.0 | 1.80 ± 0.4 | 0.77 ± 0.4 | 4.39 ± 2.1 | 2.16 ± 0.7 |
| **P5** | 30.56 ± 3.4 | 50.45 ± 5.8 | 53.97 ± 2.9 | 49.50 ± 6.8 | 22.06 ± 3.4 | 24.21 ± 10 | 58.63 ± 10.5 | 36.13 ± 8.2 | 1.84 ± 0.8 | 1.41 ± 1.5 | 3.87 ± 1.4 | 2.76 ± 0.7 |
| **P10** | 35.55 ± 3.5 | 47.74 ± 6.2 | 54.84 ± 4.0 | 41.52 ± 7.8 | 23.47 ± 5.2 | 32.77 ± 4.5 | 53.98 ± 7.3 | 35.55 ± 9.3 | 1.95 ± 0.5 | 0.90 ± 0.5 | 4.67 ± 1.6 | 2.46 ± 1.5 |
| **S1** | 31.34 ± 4.2 | 49.20 ± 5.9 | 52.69 ± 3.5 | 47.74 ± 2.5 | 22.72 ± 6.1 | 30.10 ± 6.9 | 52.49 ± 6.2 | 44.13 ± 7.8 | 2.24 ± 0.7 | 0.63 ± 0.3 | 4.09 ± 1.5 | 4.01 ± 2.3 |
| **S5** | 31.88 ± 2.9 | 50.55 ± 8.5 | 57.57 ± 6.2 | 46.56 ± 5.8 | 24.06 ± 4.2 | 35.76 ± 6.5 | 51.42 ± 10.7 | 35.06 ± 9.7 | 2.05 ± 0.7 | 0.67 ± 0.3 | 4.32 ± 2.5 | 2.49 ± 1.1 |
| **S10** | 37.19 ± 3.4 | 47.79 ± 5.8 | 58.35 ± 6.7 | 53.54 ± 3.0 | 21.28 ± 3.4 | 34.58 ± 4.2 | 49.94 ± 4.7 | 32.58 ± 14.3 | 1.72 ± 0.6 | 0.97 ± 0.5 | 4.90 ± 2.3 | 1.53 ± 0.1 |
| **T1** | 36.08 ± 4.0 | 48.02 ± 7.5 | 58.68 ± 6.2 | 53.34 ± 6.1 | 18.12 ± 3.8 | 32.18 ± 7.4 | 44.22 ± 13.2 | 36.48 ± 7.6 | 2.41 ± 0.9 | 1.82 ± 1.8 | 4.33 ± 3.9 | 3.68 ± 3.1 |
| **T5** | 36.00 ± 3.3 | 48.53 ± 7.3 | 55.58 ± 5.5 | 54.89 ± 8.5 | 20.06 ± 3.3 | 26.18 ± 4.5 | 48.71 ± 7.1 | 42.12 ± 13.2 | 2.04 ± 0.4 | 2.01 ± 2.2 | 4.83 ± 3.7 | 1.76 ± 0.3 |
| **T10** | 33.14 ± 3.1 | 49.26 ± 5.1 | 59.06 ± 7.3 | 55.71 ± 5.5 | 19.97 ± 3.2 | 25.99 ± 6.0 | 45.71 ± 9.7 | 27.96 ± 6.3 | 2.01 ± 0.4 | 0.92 ± 0.5 | 4.32 ± 2.5 | 1.12 ± 0.2 |
| **Carbon** | ***B. napus*** | ***C. bursa-past.*** | ***T. aestivum*** | ***A. spica-venti*** | ***B. napus*** | ***C. bursa-past.*** | ***T. aestivum*** | ***A. spica-venti*** | ***B. napus*** | ***C. bursa-past.*** | ***T. aestivum*** | ***A. spica-venti*** |
|  | Leaves | Leaves | Leaves | Leaves | Stems | Stems | Stems | Stems | Roots | Roots | Roots | Roots |
| **C** | 419.3 ± 14.2 | 354.6 ± 22.0 | 396.2 ± 51.5 | 431.0 ± 15.7 | 429.8 ± 20.2 | 419.6 ± 8.3 | 353.6 ± 47.2 | 390.0 ± 30.9 | 381.5 ± 40.3 | 459.5 ± 21.5 | 465.4 ± 15.4 | 423.8 ± 42.8 |
| **P1** | 403.1 ± 8.6 | 377.6 ± 13.5 | 387.2 ± 36.4 | 419.6 ± 18.1 | 436.0 ± 10.7 | 414.8 ± 6.4 | 361.6 ± 31.2 | 383.7 ± 31.0 | 436.1 ± 16.4 | 443.7 ± 22.5 | 448.9 ± 16.6 | 441.6 ± 27.5 |
| **P5** | 411.1 ± 9.4 | 378.1 ± 10.4 | 406.7 ± 17.7 | 421.9 ± 17.4 | 434.9 ± 8.4 | 409.0 ± 11.7 | 367.2 ± 28.9 | 382.6 ± 17.3 | 436.3 ± 12.9 | 436.6 ± 24.2 | 433.9 ± 20.0 | 406.9 ± 47.5 |
| **P10** | 404.7 ± 9.7 | 404.6 ± 14.3 | 417.9 ± 16.5 | 420.9 ± 8.5 | 423.3 ± 12.5 | 402.3 ± 33.2 | 326.3 ± 43.6 | 360.2 ± 67.6 | 427.4 ± 14.7 | 429.9 ± 20.2 | 439.6 ± 39.3 | 433.9 ± 27.2 |
| **S1** | 415.4 ± 14.7 | 379.7 ± 1.0 | 421.1 ± 12.8 | 438.3 ± 8.0 | 427.8 ± 20.1 | 420.7 ± 8.4 | 336.3 ± 43.1 | 399.1 ± 9.5 | 380.5 ± 42.9 | 436.2 ± 18.7 | 439.2 ± 23.5 | 429.4 ± 15.3 |
| **S5** | 417.9 ± 7.0 | 382.8 ± 17.2 | 372.1 ± 62.1 | 423.3 ± 14.8 | 430.6 ± 13.8 | 415.8 ± 6.5 | 327.4 ± 58.7 | 394.5 ± 15.3 | 412.6 ± 27.5 | 433.3 ± 23.8 | 447.9 ± 21.2 | 401.5 ± 47.9 |
| **S10** | 414.3 ± 9.2 | 379.2 ± 38.0 | 387.0 ± 53.5 | 423.3 ± 18.3 | 427.4 ± 7.0 | 419.4 ± 14.4 | 361.6 ± 40.8 | 403.4 ± 18.8 | 404.2 ± 20.5 | 436.1 ± 12.2 | 458.8 ± 17.3 | 380.7 ± 43.6 |
| **T1** | 409.1 ± 8.3 | 370.4 ± 11.2 | 395.4 ± 42.7 | 438.2 ± 11.5 | 417.1 ± 10.2 | 408.4 ± 14.1 | 356.9 ± 48.4 | 400.1 ± 17.0 | 399.3 ± 28.5 | 433.0 ± 38.4 | 473.9 ± 14.4 | 394.5 ± 33.1 |
| **T5** | 412.6 ± 9.6 | 370.1 ± 9.1 | 408.1 ± 23.9 | 424.1 ± 12.9 | 430.7 ± 9.0 | 420.9 ± 8.7 | 366.0 ± 22.8 | 390.4 ± 24.7 | 393.8 ± 46.2 | 440.3 ± 26.7 | 457.4 ± 13.5 | 434.0 ± 24.7 |
| **T10** | 416.6 ± 7.0 | 369.9 ± 12.8 | 398.2 ± 35.2 | 419.5 ± 27.3 | 435.9 ± 4.9 | 413.3 ± 35.3 | 350.9 ± 29.2 | 397.0 ± 28.6 | 377.4 ± 61.7 | 447.2 ± 17.8 | 459.9 ± 15.9 | 422.6 ± 30.0 |
| **Calcium** | ***B. napus*** | ***C. bursa-past.*** | ***T. aestivum*** | ***A. spica-venti*** | ***B. napus*** | ***C. bursa-past.*** | ***T. aestivum*** | ***A. spica-venti*** | ***B. napus*** | ***C. bursa-past.*** | ***T. aestivum*** | ***A. spica-venti*** |
|  | Leaves | Leaves | Leaves | Leaves | Stems | Stems | Stems | Stems | Roots | Roots | Roots | Roots |
| **C** | 28.87 ± 3.8 | 40.41 ± 5.8 | 12.35 ± 6.0 | 10.24 ± 3.9 | 9.62 ± 2.8 | 15.39 ± 3.2 | 22.07 ± 10.7 | 9.68 ± 1.7 | 21.52 ± 5.7 | 13.57 ± 1.5 | 7.55 ± 1.4 | 9.6 ± 1.4 |
| **P1** | 28.27 ± 6.9 | 41.36 ± 3.6 | 12.73 ± 5.7 | 10.43 ± 3.5 | 9.56 ± 1.8 | 14.78 ± 3.8 | 23.48 ± 7.2 | 13.33 ± 4.5 | 18.56 ± 3.8 | 14.92 ± 2.5 | 6.11 ± 0.6 | 10.63 ± 0.8 |
| **P5** | 27.97 ± 1.5 | 40.05 ± 5.3 | 12.63 ± 3.8 | 12.33 ± 5.3 | 9.45 ± 1.6 | 13.80 ± 3.4 | 18.90 ± 6.2 | 12.03 ± 4.0 | 23.69 ± 7.2 | 14.08 ± 1.3 | 6.16 ± 1.3 | 10.08 ± 1.4 |
| **P10** | 29.86 ± 4.9 | 39.62 ± 4.4 | 15.61 ± 6.7 | 12.37 ± 4.5 | 10.98 ± 2.6 | 15.75 ± 4.0 | 28.78 ± 10.0 | 13.51 ± 5.1 | 21.71 ± 5.5 | 14.30 ± 1.7 | 6.09 ± 2.0 | 8.68 ± 3.6 |
| **S1** | 28.56 ± 4.5 | 44.26 ± 5.6 | 11.03 ± 2.7 | 9.75 ± 0.7 | 10.11 ± 3.4 | 15.79 ± 3.7 | 25.41 ± 11.8 | 9.66 ± 2.1 | 19.06 ± 6.4 | 15.28 ± 1.1 | 7.23 ± 1.3 | 10.5 ± 0.9 |
| **S5** | 30.35 ± 4.3 | 39.90 ± 5.1 | 14.55 ± 8.3 | 11.80 ± 2.7 | 9.02 ± 2.5 | 15.02 ± 2.6 | 25.35 ± 15.0 | 14.24 ± 4.1 | 17.01 ± 4.7 | 14.81 ± 1.1 | 6.52 ± 0.9 | 10.31 ± 1.0 |
| **S10** | 28.40 ± 2.9 | 41.51 ± 4.6 | 12.66 ± 6.3 | 11.13 ± 2.5 | 9.95 ± 1.4 | 15.80 ± 5.1 | 19.63 ± 11.0 | 10.30 ± 4.1 | 27.93 ± 4.9 | 14.95 ± 1.9 | 7.55 ± 1.7 | 10.17 ± 1.0 |
| **T1** | 28.20 ± 4.2 | 42.42 ± 2.3 | 11.02 ± 4.1 | 8.95 ± 1.2 | 9.97 ± 2.6 | 18.43 ± 2.9 | 20.02 ± 12.7 | 8.53 ± 1.3 | 20.97 ± 11.0 | 14.22 ± 1.4 | 6.88 ± 1.5 | 9.25 ± 1.1 |
| **T5** | 29.96 ± 3.4 | 44.43 ± 4.8 | 11.69 ± 3.7 | 11.49 ± 2.4 | 9.86 ± 1.7 | 18.50 ± 1.4 | 21.3 ± 10.3 | 11.93 ± 4.1 | 17.48 ± 4.2 | 13.06 ± 2.5 | 7.76 ± 2.4 | 8.88 ± 0.6 |
| **T10** | 30.14 ± 3.1 | 42.55 ± 3.1 | 12.96 ± 5.4 | 11.64 ± 4.3 | 9.26 ± 1.1 | 15.86 ± 3.7 | 19.89 ± 9.4 | 13.40 ± 8.2 | 16.33 ± 4.3 | 13.91 ± 2.7 | 7.74 ± 1.6 | 8.5 ± 1.4 |
| **Sulfur** | ***B. napus*** | ***C. bursa-past.*** | ***T. aestivum*** | ***A. spica-venti*** | ***B. napus*** | ***C. bursa-past.*** | ***T. aestivum*** | ***A. spica-venti*** | ***B. napus*** | ***C. bursa-past.*** | ***T. aestivum*** | ***A. spica-venti*** |
|  | Leaves | Leaves | Leaves | Leaves | Stems | Stems | Stems | Stems | Roots | Roots | Roots | Roots |
| **C** | 10.26 ± 2.2 | 7.74 ± 0.7 | 3.92 ± 0.7 | 3.46 ± 0.3 | 2.80 ± 1.2 | 7.39 ± 1.7 | 10.48 ± 5.4 | 3.47 ± 0.9 | 2.55 ± 0.5 | 2.67 ± 0.5 | 1.86 ± 0.5 | 1.97 ± 0.4 |
| **P1** | 10.79 ± 3.0 | 6.95 ± 0.3 | 3.72 ± 0.7 | 3.89 ± 0.2 | 3.16 ± 0.5 | 7.04 ± 1.0 | 12.32 ± 3.6 | 5.35 ± 2.4 | 2.15 ± 0.4 | 2.94 ± 0.4 | 1.50 ± 0.5 | 2.32 ± 0.4 |
| **P5** | 9.10 ± 1.6 | 7.30 ± 0.5 | 3.74 ± 0.6 | 4.19 ± 0.7 | 3.16 ± 0.7 | 6.47 ± 0.8 | 10.68 ± 3.3 | 4.15 ± 1.3 | 2.30 ± 0.6 | 2.89 ± 0.4 | 1.56 ± 0.5 | 2.18 ± 0.3 |
| **P10** | 10.54 ± 1.9 | 7.22 ± 0.9 | 4.30 ± 0.5 | 3.85 ± 0.4 | 4.10 ± 1.6 | 7.10 ± 0.4 | 13.31 ± 5.4 | 4.00 ± 2.2 | 1.91 ± 0.2 | 2.81 ± 0.3 | 1.67 ± 1.3 | 1.82 ± 0.5 |
| **S1** | 9.49 ± 2.3 | 7.55 ± 0.7 | 4.26 ± 0.5 | 4.11 ± 0.2 | 3.92 ± 1.5 | 6.25 ± 1.1 | 12.29 ± 6.4 | 3.53 ± 0.8 | 1.70 ± 0.2 | 2.65 ± 0.4 | 1.93 ± 0.6 | 2.45 ± 0.4 |
| **S5** | 11.06 ± 2.0 | 7.22 ± 0.7 | 3.87 ± 0.4 | 4.03 ± 0.4 | 3.94 ± 0.6 | 7.00 ± 1.4 | 12.09 ± 7.6 | 4.99 ± 2.5 | 2.21 ± 0.5 | 2.79 ± 0.3 | 1.83 ± 0.5 | 2.07 ± 0.5 |
| **S10** | 10.03 ± 1.5 | 7.34 ± 1.3 | 3.99 ± 0.6 | 3.44 ± 0.5 | 2.79 ± 0.2 | 7.89 ± 2.3 | 10.60 ± 5.4 | 3.20 ± 2.8 | 2.02 ± 0.2 | 2.67 ± 0.5 | 2.39 ± 1.0 | 1.50 ± 0.6 |
| **T1** | 9.47 ± 2.7 | 7.36 ± 0.6 | 4.05 ± 0.3 | 3.30 ± 0.2 | 3.47 ± 1.7 | 6.71 ± 1.1 | 10.49 ± 6.7 | 2.28 ± 0.4 | 2.12 ± 0.6 | 2.92 ± 0.6 | 1.86 ± 1.0 | 1.38 ± 0.4 |
| **T5** | 11.49 ± 2.0 | 8.00 ± 1.2 | 4.24 ± 0.2 | 3.55 ± 0.3 | 3.66 ± 0.8 | 6.51 ± 0.9 | 12.43 ± 7.5 | 3.82 ± 1.8 | 2.13 ± 0.5 | 2.67 ± 0.5 | 1.69 ± 0.9 | 1.69 ± 0.3 |
| **T10** | 11.34 ± 1.1 | 7.37 ± 0.7 | 4.17 ± 0.6 | 3.67 ± 0.4 | 3.41 ± 0.6 | 6.40 ± 1.0 | 9.27 ± 4.4 | 4.21 ± 3.1 | 1.72 ± 0.6 | 2.49 ± 0.5 | 1.88 ± 0.6 | 1.30 ± 0.3 |
| **Copper** | ***B. napus*** | ***C. bursa-past.*** | ***T. aestivum*** | ***A. spica-venti*** | ***B. napus*** | ***C. bursa-past.*** | ***T. aestivum*** | ***A. spica-venti*** | ***B. napus*** | ***C. bursa-past.*** | ***T. aestivum*** | ***A. spica-venti*** |
|  | Leaves | Leaves | Leaves | Leaves | Stems | Stems | Stems | Stems | Roots | Roots | Roots | Roots |
| **C** | 0.017 ± 0.002 | 0.013 ± 0.001 | 0.013 ± 0.004 | 0.019 ± 0.002 | NA | NA | 0.046 ± 0.034 | 0.032 ± 0.009 | 0.247 ± 0.128 | 0.582 ± 0.257 | 0.599 ± 0.301 | 0.691 ± 0.249 |
| **P1** | 0.016 ± 0.002 | NA | 0.016 ± 0.006 | 0.019 ± 0.003 | NA | 0.037 ± 0.006 | 0.055 ± 0.015 | 0.043 ± 0.022 | 0.385 ± 0.078 | 0.515 ± 0.425 | 0.542 ± 0.221 | 0.879 ± 0.259 |
| **P5** | 0.015 ± 0.002 | NA | 0.016 ± 0.003 | 0.018 ± 0.005 | NA | NA | 0.057 ± 0.010 | 0.037 ± 0.011 | 0.414 ± 0.122 | 0.408 ± 0.286 | 0.674 ± 0.287 | 0.517 ± 0.124 |
| **P10** | 0.016 ± 0.002 | NA | 0.019 ± 0.013 | 0.020 ± 0.005 | NA | NA | 0.057 ± 0.029 | 0.030 ± 0.008 | 0.293 ± 0.052 | 0.418 ± 0.454 | 0.574 ± 0.256 | 0.660 ± 0.291 |
| **S1** | 0.017 ± 0.003 | NA | 0.013 ± 0.002 | 0.017 ± 0.004 | NA | NA | 0.052 ± 0.030 | 0.028 ± 0.01 | 0.308 ± 0.082 | 0.625 ± 0.154 | 0.444 ± 0.212 | 0.768 ± 0.270 |
| **S5** | 0.015 ± 0.001 | NA | 0.014 ± 0.001 | 0.018 ± 0.001 | NA | NA | 0.086 ± 0.032 | 0.030 ± 0.008 | 0.429 ± 0.286 | 0.425 ± 0.228 | 0.372 ± 0.193 | 0.674 ± 0.216 |
| **S10** | 0.019 ± 0.002 | NA | 0.012 ± 0.001 | 0.022 ± 0.004 | NA | 0.017 ± 0.008 | 0.060 ± 0.023 | 0.037 ± 0.033 | 0.357 ± 0.110 | 0.565 ± 0.243 | 0.349 ± 0.188 | 0.698 ± 0.120 |
| **T1** | 0.018 ± 0.003 | NA | 0.011 ± 0.001 | 0.020 ± 0.002 | NA | NA | 0.047 ± 0.039 | 0.035 ± 0.005 | 0.303 ± 0.169 | 0.614 ± 0.185 | 0.539 ± 0.365 | 0.471 ± 0.348 |
| **T5** | 0.020 ± 0.003 | NA | 0.013 ± 0.002 | 0.020 ± 0.002 | NA | NA | 0.040 ± 0.011 | NA | 0.284 ± 0.079 | 0.577 ± 0.288 | 0.443 ± 0.306 | 0.461 ± 0.221 |
| **T10** | 0.019 ± 0.003 | NA | 0.012 ± 0.003 | 0.019 ± 0.001 | NA | NA | 0.039 ± 0.020 | 0.031 ± 0.008 | 0.248 ± 0.076 | 0.455 ± 0.315 | 0.385 ± 0.095 | 0.379 ± 0.085 |
|  |  |  |  |  |  |  |  |  |  |  |  |  |
|  |  |  |  |  |  |  |  |  |  |  |  |  |
|  |  |  |  |  |  |  |  |  |  |  |  |  |
|  |  |  |  |  |  |  |  |  |  |  |  |  |
|  |  |  |  |  |  |  |  |  |  |  |  |  |
|  |  |  |  |  |  |  |  |  |  |  |  |  |
|  |  |  |  |  |  |  |  |  |  |  |  |  |
|  |  |  |  |  |  |  |  |  |  |  |  |  |
| *Appendix 3 continued* | | | | | |  |  |  |  |  |  |  |
| **Magnesium** | ***B. napus*** | ***C. bursa-past.*** | ***T. aestivum*** | ***A. spica-venti*** | ***B. napus*** | ***C. bursa-past.*** | ***T. aestivum*** | ***A. spica-venti*** | ***B. napus*** | ***C. bursa-past.*** | ***T. aestivum*** | ***A. spica-venti*** |
|  | Leaves | Leaves | Leaves | Leaves | Stems | Stems | Stems | Stems | Roots | Roots | Roots | Roots |
| **C** | 6.69 ± 0.9 | 9.55 ± 1.9 | 6.64 ± 4.1 | 5.16 ± 1.6 | 2.57 ± 0.7 | 2.75 ± 0.6 | 7.30 ± 3.2 | 3.76 ± 0.8 | 1.40 ± 0.1 | 1.79 ± 0.2 | 1.13 ± 0.3 | 1.32 ± 0.3 |
| **P1** | 6.01 ± 1.0 | 9.46 ± 0.8 | 7.56 ± 3.7 | 5.03 ± 1.5 | 2.77 ± 1.2 | 2.69 ± 0.4 | 8.20 ± 3.2 | 5.32 ± 1.8 | 1.27 ± 0.2 | 1.87 ± 0.3 | 0.81 ± 0.2 | 1.60 ± 0.2 |
| **P5** | 5.91 ± 0.6 | 8.38 ± 1.5 | 6.88 ± 2.4 | 5.55 ± 2.0 | 2.32 ± 0.5 | 2.94 ± 0.5 | 5.86 ± 2.1 | 5.47 ± 1.4 | 1.38 ± 0.2 | 1.97 ± 0.2 | 0.74 ± 0.2 | 1.75 ± 0.2 |
| **P10** | 6.28 ± 0.4 | 8.83 ± 0.5 | 7.35 ± 3.9 | 5.64 ± 1.4 | 2.94 ± 0.6 | 2.59 ± 0.4 | 9.09 ± 2.9 | 7.09 ± 2.2 | 1.28 ± 0.1 | 1.99 ± 0.3 | 0.78 ± 0.2 | 1.58 ± 0.7 |
| **S1** | 6.35 ± 0.8 | 9.45 ± 1.3 | 5.27 ± 1.5 | 4.41 ± 0.4 | 2.72 ± 0.9 | 2.21 ± 0.3 | 8.49 ± 2.7 | 4.74 ± 0.9 | 1.19 ± 0.2 | 1.73 ± 0.2 | 0.98 ± 0.3 | 2.09 ± 0.6 |
| **S5** | 6.58 ± 0.7 | 8.18 ± 0.5 | 7.82 ± 5.2 | 5.58 ± 1.3 | 2.62 ± 0.8 | 2.51 ± 0.7 | 8.96 ± 3.7 | 6.26 ± 1.3 | 1.20 ± 0.1 | 2.04 ± 0.4 | 1.04 ± 0.3 | 1.67 ± 0.4 |
| **S10** | 6.44 ± 0.6 | 8.21 ± 0.6 | 6.57 ± 4.4 | 5.56 ± 1.6 | 2.94 ± 0.3 | 2.75 ± 0.8 | 6.63 ± 2.7 | 4.06 ± 0.5 | 1.51 ± 0.1 | 1.88 ± 0.1 | 1.15 ± 0.3 | 1.40 ± 0.1 |
| **T1** | 6.55 ± 0.8 | 8.41 ± 1.2 | 4.83 ± 2.4 | 4.38 ± 0.8 | 3.05 ± 0.6 | 2.42 ± 0.2 | 6.51 ± 3.3 | 4.32 ± 0.6 | 1.38 ± 0.3 | 1.82 ± 0.2 | 0.92 ± 0.2 | 1.79 ± 0.4 |
| **T5** | 6.32 ± 0.8 | 9.72 ± 1.5 | 5.73 ± 2.4 | 5.75 ± 1.7 | 2.84 ± 0.5 | 2.50 ± 0.5 | 6.04 ± 1.8 | 5.30 ± 1.3 | 1.18 ± 0.1 | 1.66 ± 0.1 | 1.15 ± 0.3 | 1.48 ± 0.2 |
| **T10** | 6.23 ± 0.6 | 9.21 ± 0.9 | 6.23 ± 3.2 | 6.21 ± 2.9 | 2.54 ± 0.4 | 2.71 ± 0.4 | 7.65 ± 3.2 | 4.67 ± 2.4 | 1.08 ± 0.1 | 1.81 ± 0.4 | 1.17 ± 0.4 | 1.14 ± 0.1 |
| **Iron** | ***B. napus*** | ***C. bursa-past.*** | ***T. aestivum*** | ***A. spica-venti*** | ***B. napus*** | ***C. bursa-past.*** | ***T. aestivum*** | ***A. spica-venti*** | ***B. napus*** | ***C. bursa-past.*** | ***T. aestivum*** | ***A. spica-venti*** |
|  | Leaves | Leaves | Leaves | Leaves | Stems | Stems | Stems | Stems | Roots | Roots | Roots | Roots |
| **C** | 0.14 ± 0.07 | 0.35 ± 0.21 | 0.13 ± 0.13 | 0.23 ± 0.10 | 0.03 ± 0.01 | 0.10 ± 0.02 | 0.71 ± 0.74 | 4.60 ± 3.53 | 4.55 ± 0.82 | 5.13 ± 2.79 | 5.90 ± 2.21 | 9.26 ± 1.51 |
| **P1** | 0.10 ± 0.02 | 0.16 ± 0.04 | 0.52 ± 0.40 | 0.13 ± 0.02 | 0.03 ± 0.01 | 0.12 ± 0.04 | 0.33 ± 0.08 | 4.54 ± 5.38 | 2.39 ± 0.48 | 3.50 ± 1.50 | 7.61 ± 2.83 | 12.21 ± 2.25 |
| **P5** | 0.10 ± 0.01 | 0.17 ± 0.06 | 0.62 ± 0.60 | 0.13 ± 0.03 | 0.04 ± 0.02 | 0.64 ± 1.26 | 0.38 ± 0.18 | 4.36 ± 2.36 | 2.66 ± 0.72 | 3.39 ± 0.89 | 8.52 ± 2.28 | 10.37 ± 3.96 |
| **P10** | 0.13 ± 0.11 | 0.31 ± 0.37 | 0.36 ± 0.38 | 0.18 ± 0.12 | 0.03 ± 0.01 | 0.09 ± 0.05 | 0.53 ± 0.25 | 2.06 ± 2.86 | 3.28 ± 1.43 | 4.30 ± 1.55 | 7.76 ± 2.15 | 10.71 ± 3.64 |
| **S1** | 0.11 ± 0.09 | 0.16 ± 0.04 | 0.07 ± 0.01 | 0.14 ± 0.02 | 0.05 ± 0.02 | 0.11 ± 0.06 | 0.46 ± 0.27 | 1.78 ± 2.42 | 5.47 ± 2.86 | 4.23 ± 2.02 | 5.92 ± 1.87 | 8.28 ± 2.05 |
| **S5** | 0.09 ± 0.01 | 0.17 ± 0.03 | 0.12 ± 0.06 | 0.15 ± 0.05 | 0.02 ± 0.01 | 0.10 ± 0.04 | 0.64 ± 0.35 | 1.97 ± 1.95 | 5.77 ± 3.41 | 3.78 ± 1.04 | 6.13 ± 2.14 | 9.25 ± 1.37 |
| **S10** | 0.14 ± 0.16 | 0.25 ± 0.22 | 0.10 ± 0.05 | 0.14 ± 0.03 | 0.03 ± 0.01 | 0.16 ± 0.11 | 0.45 ± 0.28 | 3.93 ± 4.82 | 3.95 ± 1.20 | 5.85 ± 4.60 | 5.44 ± 2.40 | 10.47 ± 0.27 |
| **T1** | 0.09 ± 0.02 | 0.14 ± 0.02 | 0.09 ± 0.02 | 0.13 ± 0.02 | 0.04 ± 0.02 | 0.13 ± 0.08 | 0.73 ± 0.69 | 1.53 ± 1.99 | 4.20 ± 0.84 | 5.21 ± 3.28 | 5.99 ± 2.25 | 7.97 ± 2.44 |
| **T5** | 0.27 ± 0.31 | 0.40 ± 0.25 | 0.16 ± 0.12 | 0.14 ± 0.02 | 0.03 ± 0.01 | 0.19 ± 0.20 | 0.37 ± 0.16 | 0.95 ± 1.39 | 5.03 ± 1.29 | 5.31 ± 1.19 | 8.41 ± 5.16 | 8.00 ± 2.16 |
| **T10** | 0.09 ± 0.01 | 0.19 ± 0.14 | 0.17 ± 0.22 | 0.18 ± 0.08 | 0.04 ± 0.01 | 0.23 ± 0.34 | 0.33 ± 0.18 | 2.42 ± 2.64 | 3.89 ± 1.45 | 4.50 ± 1.74 | 6.26 ± 2.14 | 8.10 ± 2.41 |
| **Manganese** | ***B. napus*** | ***C. bursa-past.*** | ***T. aestivum*** | ***A. spica-venti*** | ***B. napus*** | ***C. bursa-past.*** | ***T. aestivum*** | ***A. spica-venti*** | ***B. napus*** | ***C. bursa-past.*** | ***T. aestivum*** | ***A. spica-venti*** |
|  | Leaves | Leaves | Leaves | Leaves | Stems | Stems | Stems | Stems | Roots | Roots | Roots | Roots |
| **C** | 0.10 ± 0.02 | 0.16 ± 0.03 | 0.17 ± 0.04 | 0.20 ± 0.05 | 0.01 ± 0.002 | 0.05 ± 0.02 | 0.15 ± 0.04 | 0.23 ± 0.05 | 0.13 ± 0.03 | 0.18 ± 0.04 | 0.35 ± 0.15 | 0.53 ± 0.26 |
| **P1** | 0.11 ± 0.02 | 0.16 ± 0.02 | 0.15 ± 0.05 | 0.24 ± 0.05 | 0.01 ± 0.007 | 0.04 ± 0.02 | 0.18 ± 0.06 | 0.31 ± 0.06 | 0.1 ± 0.02 | 0.16 ± 0.03 | 0.50 ± 0.20 | 0.65 ± 0.46 |
| **P5** | 0.10 ± 0.03 | 0.15 ± 0.04 | 0.18 ± 0.03 | 0.29 ± 0.12 | 0.01 ± 0.005 | 0.04 ± 0.01 | 0.17 ± 0.04 | 0.32 ± 0.06 | 0.12 ± 0.03 | 0.14 ± 0.05 | 0.45 ± 0.17 | 0.51 ± 0.18 |
| **P10** | 0.10 ± 0.02 | 0.20 ± 0.04 | 0.19 ± 0.05 | 0.29 ± 0.03 | 0.01 ± 0.006 | 0.05 ± 0.01 | 0.18 ± 0.04 | 0.38 ± 0.11 | 0.12 ± 0.02 | 0.21 ± 0.09 | 0.38 ± 0.17 | 0.49 ± 0.18 |
| **S1** | 0.09 ± 0.02 | 0.20 ± 0.04 | 0.18 ± 0.06 | 0.24 ± 0.01 | 0.01 ± 0.004 | 0.04 ± 0.01 | 0.19 ± 0.05 | 0.31 ± 0.07 | 0.12 ± 0.04 | 0.18 ± 0.03 | 0.63 ± 0.17 | 0.60 ± 0.34 |
| **S5** | 0.11 ± 0.02 | 0.16 ± 0.03 | 0.19 ± 0.08 | 0.29 ± 0.10 | 0.01 ± 0.003 | 0.04 ± 0.01 | 0.17 ± 0.04 | 0.35 ± 0.08 | 0.13 ± 0.03 | 0.14 ± 0.03 | 0.45 ± 0.10 | 0.80 ± 0.35 |
| **S10** | 0.08 ± 0.01 | 0.21 ± 0.04 | 0.20 ± 0.03 | 0.26 ± 0.05 | 0.01 ± 0.002 | 0.06 ± 0.01 | 0.18 ± 0.03 | 0.35 ± 0.07 | 0.12 ± 0.01 | 0.26 ± 0.10 | 0.56 ± 0.13 | 0.57 ± 0.18 |
| **T1** | 0.07 ± 0.01 | 0.17 ± 0.03 | 0.16 ± 0.06 | 0.19 ± 0.03 | 0.01 ± 0.015 | 0.05 ± 0.02 | 0.13 ± 0.04 | 0.28 ± 0.07 | 0.11 ± 0.03 | 0.20 ± 0.07 | 0.36 ± 0.20 | 0.53 ± 0.21 |
| **T5** | 0.09 ± 0.03 | 0.18 ± 0.03 | 0.17 ± 0.03 | 0.22 ± 0.04 | 0.01 ± 0.003 | 0.04 ± 0.01 | 0.17 ± 0.06 | 0.25 ± 0.06 | 0.11 ± 0.01 | 0.13 ± 0.03 | 0.78 ± 0.70 | 0.41 ± 0.11 |
| **T10** | 0.10 ± 0.03 | 0.19 ± 0.03 | 0.16 ± 0.06 | 0.24 ± 0.05 | 0.01 ± 0.003 | 0.05 ± 0.01 | 0.14 ± 0.06 | 0.25 ± 0.04 | 0.09 ± 0.02 | 0.20 ± 0.06 | 0.37 ± 0.18 | 0.51 ± 0.11 |
| **Sodium** | ***B. napus*** | ***C. bursa-past.*** | ***T. aestivum*** | ***A. spica-venti*** | ***B. napus*** | ***C. bursa-past.*** | ***T. aestivum*** | ***A. spica-venti*** | ***B. napus*** | ***C. bursa-past.*** | ***T. aestivum*** | ***A. spica-venti*** |
|  | Leaves | Leaves | Leaves | Leaves | Stems | Stems | Stems | Stems | Roots | Roots | Roots | Roots |
| **C** | 5.98 ± 1.8 | 11.27 ± 8.4 | 14.97 ± 14.4 | 7.91 ± 4.4 | 8.06 ± 3.1 | 4.01 ± 1.7 | 21.67 ± 13.3 | 6.04 ± 2.5 | 1.48 ± 0.5 | 2.11 ± 0.7 | 2.88 ± 0.8 | 2.28 ± 1.0 |
| **P1** | 4.06 ± 1.3 | 4.92 ± 2.5 | 18.25 ± 11.8 | 9.13 ± 4.0 | 7.48 ± 1.9 | 4.29 ± 3.1 | 25.17 ± 8.5 | 9.78 ± 4.8 | 1.28 ± 0.3 | 1.44 ± 0.3 | 4.08 ± 1.6 | 2.97 ± 0.8 |
| **P5** | 5.81 ± 2.9 | 5.15 ± 1.9 | 13.86 ± 6.9 | 8.81 ± 4.5 | 8.01 ± 2.7 | 4.95 ± 3.2 | 18.98 ± 7.7 | 9.05 ± 4.3 | 1.35 ± 0.3 | 1.79 ± 0.7 | 3.47 ± 0.6 | 3.94 ± 0.7 |
| **P10** | 6.39 ± 1.5 | 5.07 ± 3.0 | 16.5 ± 14.2 | 10.82 ± 4.7 | 9.07 ± 2.4 | 5.22 ± 2.5 | 30.44 ± 11.3 | 16.10 ± 8.2 | 1.22 ± 0.2 | 1.68 ± 0.6 | 3.91 ± 0.8 | 3.46 ± 2.2 |
| **S1** | 5.27 ± 2.5 | 5.23 ± 3.1 | 9.05 ± 5.0 | 6.55 ± 1.3 | 7.80 ± 2.7 | 4.76 ± 3.5 | 23.51 ± 11.0 | 7.26 ± 1.8 | 1.16 ± 0.2 | 1.35 ± 0.3 | 3.76 ± 0.6 | 4.61 ± 1.7 |
| **S5** | 4.10 ± 1.1 | 4.29 ± 3.0 | 19.53 ± 17.0 | 10.48 ± 4.0 | 5.91 ± 1.4 | 4.68 ± 2.0 | 27.23 ± 16.0 | 10.86 ± 2.8 | 1.28 ± 0.1 | 1.64 ± 0.6 | 4.10 ± 1.2 | 3.53 ± 1.0 |
| **S10** | 7.55 ± 1.7 | 4.56 ± 2.1 | 14.6 ± 15.5 | 7.45 ± 2.3 | 9.33 ± 1.8 | 3.32 ± 1.7 | 17.59 ± 11.3 | 7.70 ± 4.9 | 1.34 ± 0.3 | 1.59 ± 0.4 | 4.30 ± 0.8 | 2.38 ± 0.2 |
| **T1** | 9.20 ± 4.7 | 5.16 ± 2.7 | 7.75 ± 7.9 | 5.59 ± 2.1 | 9.40 ± 3.4 | 6.00 ± 3.9 | 17.55 ± 12.2 | 8.81 ± 8.2 | 1.33 ± 0.4 | 1.96 ± 0.4 | 3.29 ± 1.0 | 4.00 ± 1.5 |
| **T5** | 5.49 ± 1.4 | 5.10 ± 3.2 | 9.91 ± 7.7 | 9.69 ± 3.3 | 6.27 ± 1.3 | 7.77 ± 9.3 | 16.62 ± 7.4 | 10.49 ± 6.0 | 1.03 ± 0.2 | 1.86 ± 0.5 | 4.32 ± 2.3 | 2.65 ± 0.4 |
| **T10** | 5.32 ± 1.7 | 5.03 ± 2.0 | 14.03 ± 12.6 | 10.32 ± 8.8 | 5.99 ± 1.3 | 5.98 ± 3.4 | 20.12 ± 8.9 | 8.22 ± 7.4 | 0.92 ± 0.2 | 1.61 ± 0.6 | 4.11 ± 0.8 | 1.61 ± 0.3 |
